# Brainstem *Dbh*^+^ neurons control allergen-induced airway hyperreactivity

**DOI:** 10.1038/s41586-024-07608-5

**Published:** 2024-07-10

**Authors:** Yujuan Su, Jinhao Xu, Ziai Zhu, Jisun Chin, Le Xu, Haoze Yu, Victoria Nudell, Barsha Dash, Esteban A. Moya, Li Ye, Axel Nimmerjahn, Xin Sun

**Affiliations:** 1 Department of Pediatrics, School of Medicine, University of California San Diego, La Jolla, CA USA; 2 Department of Biological Sciences, University of California San Diego, La Jolla, CA USA; 3 Department of Neuroscience, Scripps Research Institute, La Jolla, CA USA; 4 La Jolla Institute for Immunology, La Jolla, CA USA; 5 Division of Pulmonary, Critical Care & Sleep Medicine, Department of Medicine, University of California, San Diego, CA USA; 6 Waitt Advanced Biophotonics Center, The Salk Institute for Biological Studies, La Jolla, CA USA

**Keywords:** Peripheral nervous system, Neurophysiology

## Abstract

Exaggerated airway constriction triggered by repeated exposure to allergen, also called hyperreactivity, is a hallmark of asthma. Whereas vagal sensory neurons are known to function in allergen-induced hyperreactivity^1–3^, the identity of downstream nodes remains poorly understood. Here we mapped a full allergen circuit from the lung to the brainstem and back to the lung. Repeated exposure of mice to inhaled allergen activated the nuclei of solitary tract (nTS) neurons in a mast cell-, interleukin-4 (IL-4)- and vagal nerve-dependent manner. Single-nucleus RNA sequencing, followed by RNAscope assay at baseline and allergen challenges, showed that a *Dbh*^*+*^ nTS population is preferentially activated. Ablation or chemogenetic inactivation of *Dbh*^*+*^ nTS neurons blunted hyperreactivity whereas chemogenetic activation promoted it. Viral tracing indicated that *Dbh*^*+*^ nTS neurons project to the nucleus ambiguus (NA) and that NA neurons are necessary and sufficient to relay allergen signals to postganglionic neurons that directly drive airway constriction. Delivery of noradrenaline antagonists to the NA blunted hyperreactivity, suggesting noradrenaline as the transmitter between *Dbh*^*+*^ nTS and NA. Together, these findings provide molecular, anatomical and functional definitions of key nodes of a canonical allergen response circuit. This knowledge informs how neural modulation could be used to control allergen-induced airway hyperreactivity.

## Abstract (web summary)

Mapping a full allergen circuit from the lung to the brainstem and back, repeated exposure of mice to inhaled allergen activated the nuclei of solitary tract neurons in a mast cell-, interleukin-4- and vagal nerve-dependent manner.

## Main

Interoception defines a fundamental biological process whereby the nervous system senses and responds to the inner state of the body. Many open questions remain regarding complete multisynaptic circuits that sense signals from organs, integrate them in the nervous system and respond through modulation of organ function. The lung is a prominent source of interoceptive signals. In the lung, airway constriction, triggered by exposure to irritants including allergens, is a physiological response. Following chronic exposure, allergens will trigger exacerbated constriction, also termed airway hyperreactivity, a primary asthma morbidity and mortality.

In repeated allergen-challenged mice, but not in naive mice, either vagotomy, ablation or repression of vagal neurons abolished airway hyperreactivity^1–3^. A key knowledge gap is the brainstem neurons that are important for transmitting the chronic allergen signal. Lung-innervating vagal sensory neurons project exclusively to the nucleus of the solitary tract (nTS) region of the brainstem^4–6^. Aside from the lung, nTS integrates sensory inputs from many organs^4,7,8^. Systematic analyses of nTS neuronal diversity and connectivity are critical for interrogation of functional heterogeneity and specificity. In this study, we delineated a complete interoception neural circuit composed of multiple nodes that are both necessary and sufficient for allergen-induced airway hyperreactivity, mimicking exacerbated airway constriction in asthma.

## nTS neurons were activated by allergen

**Fig. 1:**
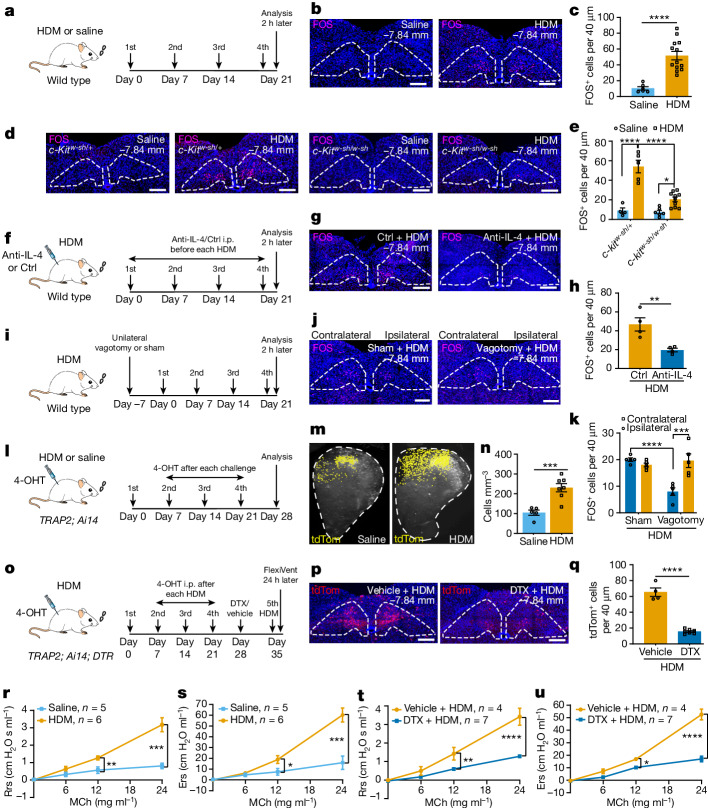
Activation of nTS neurons following repeated allergen challenges to lung. **a**, Experimental scheme for HDM treatment. **b**,**c**, Representative FOS antibody staining (**b**) and quantification (**c**). Dashed areas represent nTS. *n* = 6 saline and *n* = 13 HDM mice, unpaired *t*-test, *P* < 0.0001. **d**,**e**, Representative FOS antibody staining (**d**) and quantification (**e**), showing decreased FOS^+^ cells in the nTS of *c-Kit*^*w-sh/w-sh*^ mice, compared to *c-Kit*^*w-sh/+*^ mice. *n* = 4 saline and *n* = 5 HDM, *c-Kit*^*w-sh/+*^ mice, *P* < 0.0001; *n* = 6 saline and *n* = 10 HDM, *c-Kit*^*w-sh/w-sh*^ mice, *P* = 0.0339; for comparison between *c-Kit*^*w-sh/+*^ and *c-Kit*^*w-sh/w-sh*^ mice following HDM, *P* < 0.0001, two-way analysis of variance (ANOVA) with Bonferroni post hoc test. **f**, Experimental scheme for treatment with anti-IL-4 antibody. **g**,**h**, Representative FOS antibody staining (**g**) and quantification (**h**). *n* = 4 mice for both groups, unpaired *t*-test, *P* = 0.0095. **i**, Experimental scheme for HDM treatment after vagotomy. **j**,**k**, Representative FOS antibody staining (**j**) and quantification (**k**). *n* = 5 mice for both sham and vagotomy, *P* = 0.0001 between vagotomy ipsilateral and vagotomy contralateral; *P* < 0.0001 between sham ipsilateral and vagotomy ipsilateral; not significant (NS) for remaining pairs, two-way ANOVA (Bonferroni post hoc). **l**, Experimental scheme for labelling allergen-activated neurons in *TRAP2; Ai14* mice. **m**,**n**, Coronal view of CLARITY-cleared brainstem hemisphere (**m**, dashed areas) and quantification (**n**, *n* = 5 saline and *n* = 7 HDM mice, unpaired *t*-test, *P* = 0.0010). **o**, Experimental scheme for ablating allergen-activated neurons in *TRAP2; Ai14; DTR* mice. **p**,**q**, Representative FOS antibody staining (**p**) and quantification (**q**). *n* = 4 vehicle and *n* = 7 DTX mice, unpaired *t*-test, *P* < 0.0001. **r**,**s**, FlexiVent-measured maximal resistance (Rrs, cm H_2_O s ml^−1^, airway pressure (cm H_2_O) per time derivative of tidal volume, **r**) and elastance (Ers, cm H_2_O ml^−1^, airway pressure per tidal volume, **s**) of wild-type airways following increasing doses of methacholine (MCh), demonstrating hyperreactivity following HDM. *n* = 5 saline and *n* = 6 HDM mice, unpaired *t*-test was performed at each MCh concentration separately, for Rrs (**r**), at 12 mg ml^−1^ MCh, *P* = 0.0087; at 24 mg ml^−1^, *P* = 0.0007; for Ers (**s**), at 12 mg ml^−1^, *P* = 0.0450; and at 24 mg ml^−1^, *P* = 0.0009; NS for all other pairwise comparisons. **t**,**u**, Blunted airway hyperreactivity in DTX + HDM group. *n* = 4 vehicle and *n* = 7 DTX mice, unpaired *t*-test, for Rrs (**t**), at 12 mg ml^−1^, *P* = 0.0069; for Ers (**u**), at 12 mg ml^−1^, *P* = 0.0436; *P* < 0.0001 for both Rrs (**t**) and Ers (**u**) at 24 mg ml^−1^; NS for all other pairs. Data are presented as mean ± s.e.m., two-sided for unpaired *t*-test. **b**,**d**,**g**,**j**,**p**, Scale bars, 200 µm. Ctrl, control; i.p. intraperitoneal. Source Data

**Extended Data Fig. 1:**
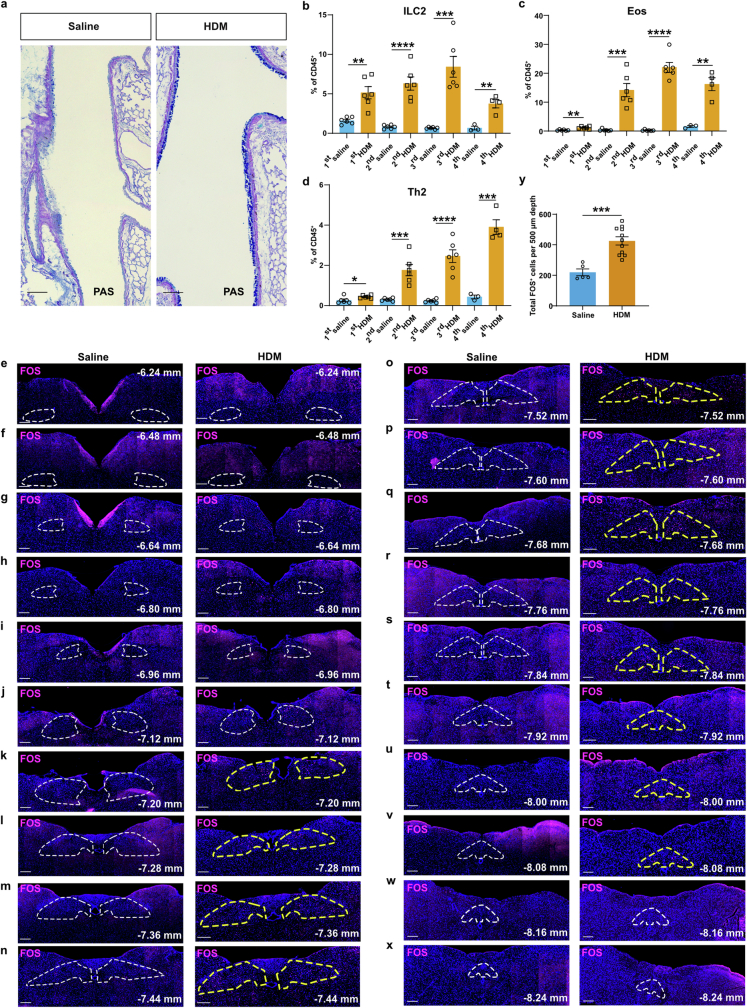
nTS neurons were activated after allergen challenge to lung. (**a**) PAS staining shows that HDM challenge effectively induced mucus-secreting airway goblet cells (blue), as expected. (**b**-**d**) Flow cytometry analyses of innate lymphoid cells (ILC2s), eosinophils (Eos) and T-helper type 2 cells (Th2) from whole lungs after consecutive challenges showing significant increase following HDM, when compared to saline controls. Data are presented as mean values ± SEM. *n* = 6 (first saline, first HDM, second saline, second HDM, third saline, third HDM), 3 (fourth saline) and 4 (fourth HDM) mice. Unpaired *t*-test (two-sided) was used between first saline and first HDM, second saline and second HDM, third saline and HDM, fourth saline and fourth HDM, separately. For **b**, *P* = 0.0012 (1^st^), *P* < 0.0001 (2^nd^), *P* = 0.0002 (3^rd^), *P* = 0.0052 (4^th^), for **c**, *P* = 0.0022 (1^st^), *P* = 0.0001 (2^nd^), *P* < 0.0001 (3^rd^), *P* = 0.0027 (4^th^), for **d**, *P* = 0.0369 (1^st^), *P* = 0.0004 (2^nd^), *P* < 0.0001 (3^rd^), *P* = 0.0005 (4^th^). (**e**-**x**) FOS antibody staining two hours after the fourth challenge in serial sections of the whole brainstem. Increased FOS^+^ cells were enriched in the nTS region from Bregma −7.20 to −8.08 mm in HDM-treated mice (outlined in yellow) compared to the saline-treated control mice. Outlines delineate nTS regions at indicated Bregma levels. (**y**) Quantification (unpaired *t*-test, two-sided, *P* = 0.0003) of total number of FOS^+^ cells after HDM (*n* = 11 mice) or saline (*n* = 5 mice) treatment in the whole nTS regions (Bregma −6.24 mm to −8.24 mm). Scale bars, 100 µm (**a**), 200 µm (**e-x**). Source Data

**Extended Data Fig. 2:**
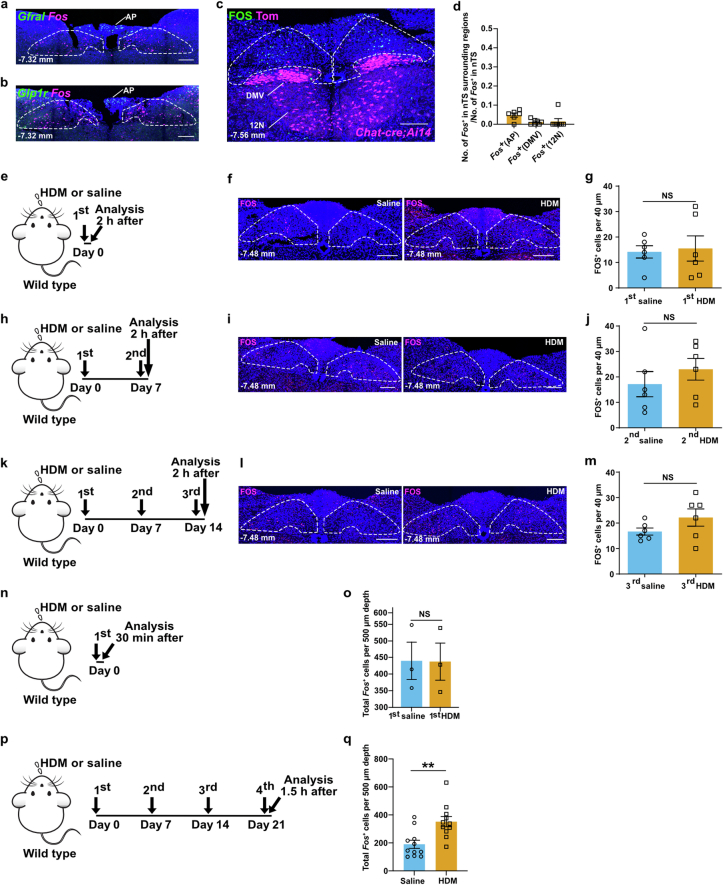
nTS neurons were activated only after repeated allergen challenges to the lung of B6 mice. (**a**,**b**) *Fos* RNAscope from brainstem section of naïve mice 1.5 h after HDM challenge to lung showing *Fos* RNA signals are not found in the AP as defined by *Gfral* (**a**) or *Glp1r* (**b**). (**c**) FOS antibody staining from brainstem section of *Chat-cre; Ai14* mice 2 h after HDM showing that there is little overlap between FOS^+^ cells and tdTom^+^ DMV and 12 N regions. (**d**) Quantifications of the number of *Fos*^*+*^ neurons in the AP (*n* = 6 mice), DMV (*n* = 7 mice) or 12 N (*n* = 7 mice). (**e**,**h**,**k**) Experiment scheme for the first, second or third HDM challenge in wild-type mice. (**f**,**g**,**i**,**j**,**l**,**m**) FOS antibody staining (**f**,**i**,**l**) and quantification (**g**,**j**,**m**) showing no statistically significant increase of FOS^+^ cells after the first, second or third HDM challenge. *n* = 6 mice each, unpaired *t*-test, NS. (**n**) Experiment scheme for single saline or HDM challenge in naive mice and tissue collection 30 min after the challenge for *Fos* RNAscope. (**o**) No significant difference in the number of *Fos*^*+*^ neurons in the nTS 30 min following a single saline (*n* = 3 mice) or HDM (*n* = 3 mice) challenge, unpaired *t*-test, NS. (**p**) Experiment scheme for HDM challenge and tissue collection in wild-type mice for *Fos* RNAscope. (**q**) The number of *Fos*^*+*^ neurons in the nTS are increased in the HDM-treated (*n* = 11 mice) group, compared to saline-treated controls (*n* = 11 mice), unpaired *t*-test, *P* = 0.0024. Outlines in **a**,**b**,**c**,**f**,**i** and **l** delineate nTS regions at indicated Bregma levels. Scale bars, 200 µm. Data are mean values ± s.e.m., statistical tests are two-sided. Source Data

To investigate the neurons involved in allergen response, we used a mouse model of asthma with chronic intranasal instillation of house dust mites (HDM), a known trigger for asthmatic responses in both humans and rodents. We used a commonly used regimen, with multiple doses of allergen administration to the lung (Fig. 1a). This triggered the expected asthmatic responses including goblet cell metaplasia, type 2 immune infiltration and exacerbated airway smooth muscle constriction, also termed airway hyperreactivity (Extended Data Fig. 1a–d and Supplementary Fig. 1a,b). To address the issue of whether allergen administered to the lung could trigger brainstem neuron activation, we performed immunostaining for immediate early-factor FOS, a neuronal activation marker. Staining at 2 h following the fourth challenge showed an increase of FOS^+^ cells in HDM-treated mice compared with saline-treated control mice (Fig. 1b,c and Extended Data Fig. 1e–y). Increased FOS^+^ cells were enriched in the nTS region between bregma −7.20 and −8.08 mm (Extended Data Fig. 1k–v) compared with the adjoining area postrema, dorsal motor nucleus of the vagus (DMV) and hypoglossal nucleus (12N) (Extended Data Fig. 2a–d). This enrichment in the nTS is consistent with findings that lung-innervating sensory nerves project to the nTS^4–6^. This increase is found to be statistically significant only after the fourth HDM challenge, but not after the previous three doses (Extended Data Fig. 2e–m), a finding corroborated by *Fos* transcript signal (Extended Data Fig. 2n–q). These findings suggest that there is conditioning of nTS neurons by repeated administration of allergen.

It is reported that mast cells are required for the development of allergen-induced airway constriction^9^. Using the same mast cell-deficient *c-Kit*^*w-sh/w-sh*^ mice as in previous studies, we found a statistically significant decrease of FOS^+^ neurons in the nTS following HDM challenges compared with heterozygous *c-Kit*^*w-sh/+*^ controls (Fig. 1d,e). These data suggest that signals relayed through mast cells are an important contributor to allergen-induced nTS activation.

Mast cells are among those immune cells that produce interleukin-4 (IL-4) and are critical for allergen-induced airway hyperreactivity^10^. It is reported that neutralization of IL-4 using antibodies can abrogate allergen-induced airway hyperreactivity^10^. Using the same IL-4-neutralizing antibody and regimen, we found that administration of anti-IL-4, but not of isotype control antibody, significantly decreased the number of FOS^+^ neurons in the nTS (Fig. 1f–h).

To determine whether vagal nerves are essential for allergen activation of the nTS, we performed unilateral vagotomy (Fig. 1i). This led to a statistically significant decrease in FOS^+^ neurons on the ipsilateral operated side compared with the contralateral control side following HDM challenge (Fig. 1j,k), suggesting that vagal nerves are required for transmission of allergen signals to the nTS.

**Extended Data Fig. 3:**
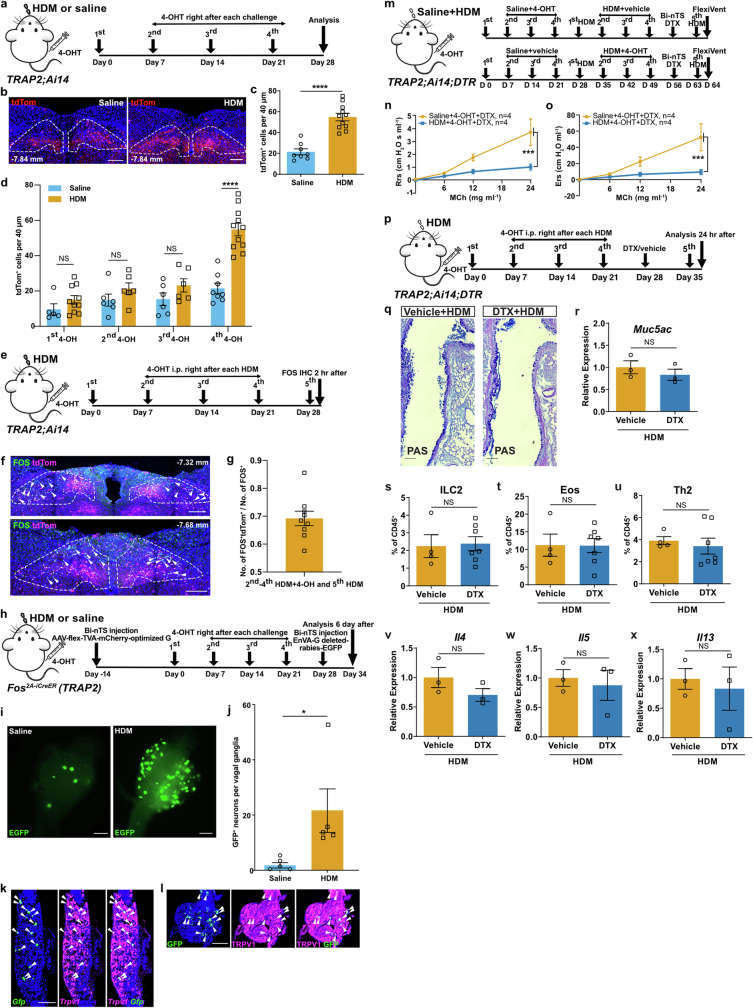
Genetic ablation of HDM-activated nTS neurons did not affect goblet cell metaplasia or immune cell infiltration. (**a**) Experiment scheme for labeling HDM-activated trapped neurons. (**b**,**c**) Representative image (**b**, nTS outlined) and quantification (**c**) from sections of *TRAP2; Ai14* mice. *n* = 8 saline and 11 HDM mice, unpaired *t*-test, *P* < 0.0001. (**d**) Quantification of tdTom^+^ nTS neurons of *TRAP2; Ai14* mice after consecutive HDM challenges. *n* = 5 saline and 10 HDM mice for first 4-OH, 6 saline and 6 HDM mice for both second and third 4-OH, 8 saline and 11 HDM mice for fourth 4-OH, unpaired *t*-test, *P* < 0.0001 fourth 4-OH saline versus fourth HDM. NS. for all other pairwise comparisons. (**e**) Experiment scheme for FOS immunostaining after trapping HDM-activated neurons. (**f**,**g**) Representative image (**f**, nTS outlined) and quantification (**g**, *n* = 9 mice) showing the overlap between FOS^+^ and tdTom^+^ nTS neurons. Arrowheads indicate tdTom^−^FOS^+^ nTS neurons. (**h**) Experiment scheme for rabies retrograde tracing of HDM-activated neurons from nTS. (**i**,**j**) Representative vagal ganglia (**i**) and quantification (**j**). *n* = 5 saline and 5 HDM mice, unpaired *t*-test, *P* = 0.0373. (**k**,**l**) Representative RNAscope (**k**) or immunostaining (**l**) images showing TRPV1^+^GFP^+^ neurons (arrowheads) in the vagal ganglia of *TRAP2* mice after rabies nTS injection and HDM challenge. (**m**) Experiment scheme for ablating saline-activated, or HDM-activated nTS neurons. (**n**,**o**) FlexiVent, *n* = 4 saline+DTX and 4 HDM + DTX mice, unpaired *t*-test, at 24 mg ml^−1^ MCh, *P* = 0.0002 for Rrs (**n**) and *P* = 0.0003 for Ers (**o**). NS for all other pairwise comparisons. (**p**) Experimental scheme for DTX ablation of HDM-activated nTS neurons. (**q**,**r**) PAS staining (**q**) and qPCR (**r**) showing no change in goblet cell metaplasia. *n* = 3 vehicle and 3 DTX mice, unpaired *t*-test, NS. (**s**-**u**) Flow cytometry analyses of ILC2s, Eos and Th2 (*n* = 4 vehicle and 7 DTX mice) and qPCR of *Il4*, *Il5* and *Il13* (*n* = 3 vehicle and 3 DTX mice), unpaired *t*-test, NS for all pairs. Scale bars, 200 µm (**b**,**f**,**i**, **k-l**), 100 µm (**q**). Data are mean ± s.e.m., statistical tests are two-sided. Source Data

To quantify cumulative allergen-induced activation and test a tool to manipulate these neurons, we crossed *Fos*^*2A-iCreER*^ (*TRAP2*) mice with *Rosa-lxl-tdTomato* (*Ai14*) mice to label activated neurons. Following injection of 4-hydroxytamoxifen (4-OHT) after each HDM challenge or saline administration (Fig. 1l), we cleared the whole brainstem using CLARITY^11^. We found a statistically significant increase of activated tdTomato^+^ (tdTom^+^) neurons in the nTS of HDM-treated mice compared with saline-treated controls (Fig. 1m,n), further confirmed through sectioning (Extended Data Fig. 3a–c). Consistently, increase in tdTom^+^ neurons is found to be statistically significant only after the fourth HDM challenge (Extended Data Fig. 3d). After performing FOS antibody staining following the fifth challenge, we found an overlap of roughly 70% with previously activated tdTom^+^ neurons (Extended Data Fig. 3e–g). This difference of around 30% may be due to either incomplete lineage labelling of activated neurons or activation of fresh new neurons in the most recent challenge.

Using *TRAP2* mice coupled with rabies virus injection in the nTS, we further found that there are more traced, mostly *Trpv1*^*+*^, neurons in the vagal ganglia of mice challenged with HDM compared with saline control (Extended Data Fig. 3h–l). This result suggests that there are enhanced synaptic connections between vagal ganglia and allergen-activated nTS neurons following HDM. Together, our data indicate that multiple allergen challenges to the lung act through immune cells and vagal ganglia to activate nTS neurons.

## Activated neurons drive hyperreactivity

To determine whether activated nTS neurons have a role in allergen-induced responses, we crossed *Fos*^*2A-iCreER*^*; Rosa-lxl-tdTomato* (*TRAP2; Ai14*) mice with *Rosa-lxl-DTR* mice to express diphtheria toxin receptor (DTR) in allergen-activated neurons (*TRAP2; Ai14; DTR*; Fig. 1o). Following bilateral nTS injection of diphtheria toxin (DTX), we confirmed a decrease in allergen-activated neurons (Fig. 1p,q).

To determine whether this ablation affects airway hyperreactivity, we utilized a well-established flexiVent assay. Following the last dose of HDM or saline, increasing doses of methacholine were intratracheally administered to mimic how acute triggers elicit chronically heightened airway constriction in humans, commonly referred to as ‘asthma attack’. In wild-type mice, as expected, multiple doses of HDM sensitization and challenge led to further increase in respiratory system resistance (Rrs) and elastance (Ers) compared with the saline control group, demonstrating allergen-induced airway hyperreactivity (Fig. 1r,s). This hyperreactivity is abrogated by vagotomy^1^, suggesting that differential airway constriction between the HDM and saline groups is primarily due to methacholine-activated vagal response rather than a direct effect of methacholine on airway smooth muscles. By comparing the extent of airway constriction triggered by the same concentration of methacholine, we used the differences between experimental and controls as a measure of the impact of the chronically adaptive vagal circuit.

In *TRAP2; Ai14; DTR* mice, compared with vehicle-injected control, DTX ablation of allergen-activated neurons led to blunted HDM-induced airway hyperreactivity (Fig. 1t,u). This blunting effect was not observed when saline-trapped (handling- and injection-activated) nTS neurons were ablated (Extended Data Fig. 3m–o). Ablation of allergen-activated neurons did not lead to any change in HDM-induced goblet cell metaplasia, type 2 immune cell recruitment or expression of type 2 cytokine genes *Il4*, *Il5* and *Il13* (Extended Data Fig. 3p–x).

## Single-nucleus transcriptome of the nTS

**Fig. 2:**
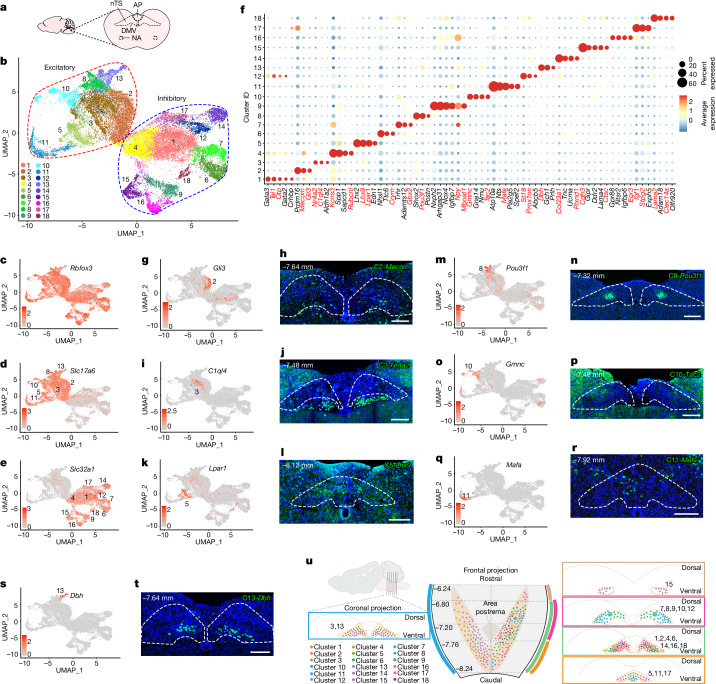
Single-nucleus transcriptomic signatures of the nTS. **a**, Diagram illustrating the relative locations of brainstem regions of interest. AP, area postrema. **b**, UMAP plots of integrated nTS snRNA-seq data from naive adult males (*n* = 4 biological repeats, *n* = 2 mice in each group), mice at 1.5 h after the fourth saline treatment (2 males in the group) and mice at 1.5 h after the fourth HDM challenge (2 males in the group). **c**–**e**, Feature plots showing pan-neuronal marker *Rbfox3* (**c**), excitatory marker *Scl17a6* (**d**) and inhibitory marker *Slc32a1* (**e**). **f**, Dotplot showing top markers for each cluster. Genes in red were used for validation by either Feature plots or RNAscope. Using default Seurat^29^ dotplot settings (Methods), percentage expressed was plotted from 0 to 60% detected and the colour bar shows the average of scaled normalized expression values across cells in a given cluster. **g**–**t**, Feature plots (**g**,**i**,**k**,**m**,**o**,**q**,**s**) and RNAscope (**h**,**j**,**l**,**n**,**p**,**r**,**t**) of excitatory markers *Gli3* (**g**), *Mecom* (**h**), *C1ql4* (**i**), *Nr4a2* (**j**), *Lpar1* (**k**), *Lhx9* (**l**), *Pou3f1* (**m**,**n**), *Gmnc* (**o**), *Tac2* (**p**), *Mafa* (**q**,**r**) and *Dbh* (**s**,**t**). Bregma levels with maximal signals are shown. Scale bars, 200 µm. **u**, Diagram summarizing spatial distribution of the 18 nTS clusters with coronal (middle) and transverse (left and right) views, based on RNAscope data on serial nTS sections. Whereas clusters 3 and 13 are found throughout the rostral–caudal axis (left), cluster 13 is ventral to cluster 3 in the rostral portion while intermingled in the caudal portion. Neurons in the other clusters are more regionally restricted to selective bregma regions, as illustrated by curved coloured lines corresponding to coloured boxes (right). Dorsal–ventral and medial–lateral distributions of each cluster are reflected by placement of coloured dots. Brainstem illustration in **u** was created with BioRender.com.

**Extended Data Fig. 4:**
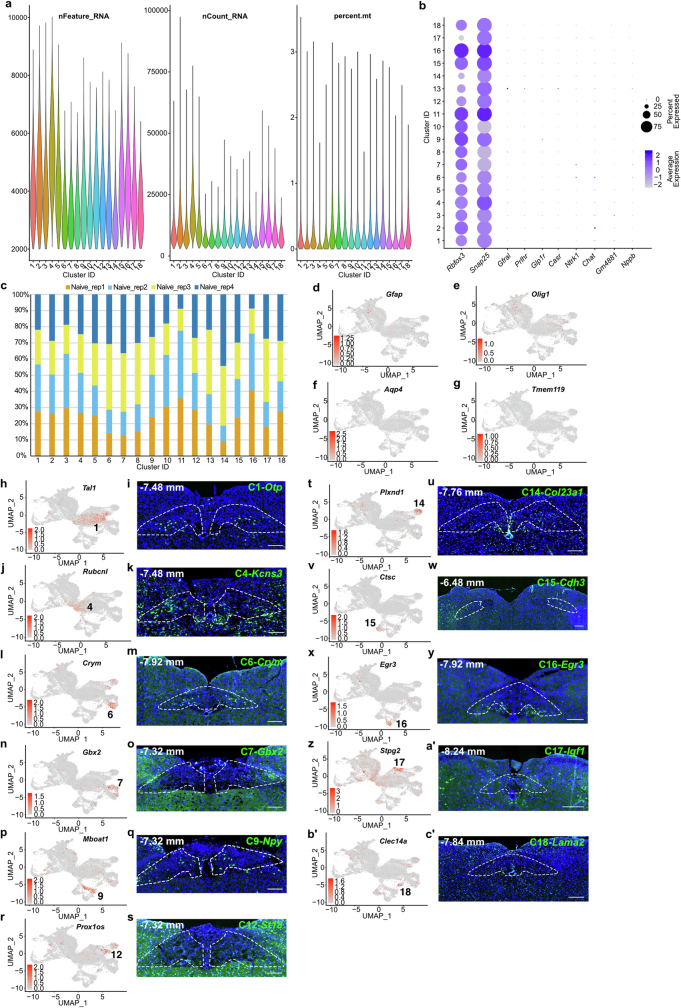
snRNA-seq provided a transcriptomic atlas of nTS. (**a**) Quality control data including nFeature RNA (the number of genes detected in each cell), nCount RNA (the total number of molecules detected within a cell) and mitochondrial percentage of our integrated nTS snRNA-seq datasets. (**b**) Dot Plot showing little expression of genes enriched in AP^15^ (*Gfral*, *Prlhr*, *Glp1r*, *Casr* and *Ntrk1*) or DMV^16^ (*Chat*, *Gm4881* and *Nppb*) compared to pan-neuronal marker genes (*Rbfox3* and *Snap25*) in our integrated nTS snRNA-seq datasets. Using default Seurat^29^ dotplot settings (Methods), percent expressed was plotted from 0 to 75% detected, and the colour bar shows the average of the scaled normalized expression values across cells in a given cluster. (**c**) Stacked bar plots showing relative contribution from four replicates of naïve nTS snRNA-seq datasets (Naïve_rep1, Naïve_rep2, Naïve_rep3 and Naïve_rep4). (**d**-**g**) Feature plots showing little to no expression of glia marker genes-*Gfap* (**d**), *Olig1* (**e**), *Aqp4* (**f**) or *Tmem119* (**g**) in the neuronal clusters. (**h**-**c**’) Feature plots (left, **h**,**j**,**l**,**n**,**p**,**r**,**t**,**v**,**x**,**z**, **b**’) and RNAscope images (right, **i**,**k**,**m**,**o**,**q**,**s**,**u**,**w**,**y**,**a**’, **c**’) showing expression of marker genes in the nTS (outlined) inhibitory clusters. Bregma levels shown are tailored to layers with optimal signal for each marker. Genes validated here by Feature plots or RNAscope were indicated in red in Fig. 2f. Scale bars, 200 µm. Source Data

To identify which subset of nTS neurons are activated, we first defined the overall diversity of nTS neurons through single nucleus RNA sequencing (snRNA-seq). Dissected and fresh-frozen nTS regions from either (1) naive mice (*n* = 4 biological repeats, 2 males in each group), (2) mice at 1.5 h following the fourth saline treatment (2 males in the group) or (3) mice at 1.5 h following the fourth HDM challenge (2 males in the group) were profiled (Fig. 2a). Following data integration from the 3 conditions, 42,157 nuclei passed quality control with more than 2,000 unique feature counts, which is a commonly used cutoff in brainstem neuronal snRNA-seq studies^12–14^. Following removal of glial and other non-neuron clusters, 39,626 neurons remained. We purified nTS neurons in silico by exclusion of known area postrema, DMV and cuneate nucleus clusters based on their snRNA-seq profiles^15,16^, as well as on the Allen Brain in situ database^17^. The resulting 32,880 nTS neurons segregated into 18 clusters, as shown by uniform manifold approximation and projection (UMAP) (Fig. 2b, Extended Data Fig. 4a,b, Supplementary Note 1, Supplementary Fig. 2a–i and Supplementary Tables 1 and 2). Following extraction of snRNA-seq data from the naive condition (Extended Data Fig. 4c), we confirmed that all 18 clusters express pan-neuronal markers but not glial markers (Fig. 2c and Extended Data Fig. 4d–g). Among the 18 neuronal clusters, 7 are excitatory and 11 are inhibitory (Fig. 2d,e). Individual neuronal populations are distinguished by a set of markers (Fig. 2f and Supplementary Table 3).

We validated the top markers using RNAscope on serial sections of the nTS (Fig. 2g–t and Extended Data Fig. 4h–c’) and generated a comprehensive map of the spatial distribution of nTS clusters (Fig. 2u). Along the rostral–caudal axis, most clusters are concentrated between bregma −6.80 and −8.24 mm. Some clusters are found in discrete domains—for example, clusters 5, 11 and 17 are found only in the more caudal regions between bregma −7.76 and −8.24 mm whereas cluster 15 is predominantly in the more rostral regions between bregma −6.24 and −6.80 mm. In comparison, other clusters—for example, clusters 3 and 13—are distributed widely along the rostral–caudal axis.

## *Dbh*^+^ neurons are preferentially activated

**Fig. 3:**
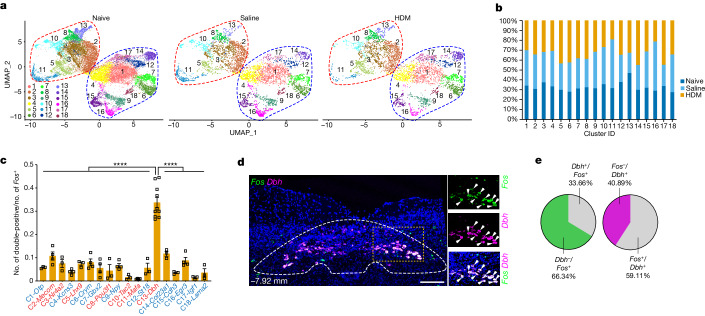
*Dbh*^*+*^ neurons in the nTS were preferentially activated following allergen challenge to lung. **a**,**b**, UMAP plots (**a**) and stacked bar plot (**b**) showing the corresponding 18 neuron clusters in naive (*n* = 4 biological repeats, *n* = 2 mice in each group), saline- (2 males in the group) and HDM-challenged (2 males in the group) nTS. **c**, Quantification from double RNAscope showing the overlap between *Fos* and the top marker gene of each individual nTS cluster 1.5 h following the fourth HDM. Red and blue indicate excitatory and inhibitory clusters, respectively. Data are mean ± s.e.m. Each data point represents an individual animal. *n* = 3 for cluster 1 (C1), *n* = 4 for C2, *n* = 3 for C3, *n* = 4 for C4, *n* = 3 for C5, *n* = 4 for C6, *n* = 4 for C7, *n* = 3 for C8, *n* = 4 for C9, *n* = 3 for C10, *n* = 4 for C11, *n* = 3 for C12, *n* = 10 for C13, *n* = 3 for C14, *n* = 3 for C15, *n* = 4 for C16, *n* = 3 for C17, *n* = 3 for C18. Multiple-comparisons one-way ANOVA (Bonferroni post hoc test), *P* < 0.0001 for comparison between C13 *Fos* and *Dbh* overlap and that between *Fos* and marker genes of the other 17 nTS clusters. **d**,**e**, *Dbh* and *Fos* double RNAscope (**d**) and quantification (*n* = 12 mice, **e**) in nTS; boxed areas are enlarged on the right, arrowheads indicate overlapping expression. Scale bars, 200 µm. Source Data

**Extended Data Fig. 5:**
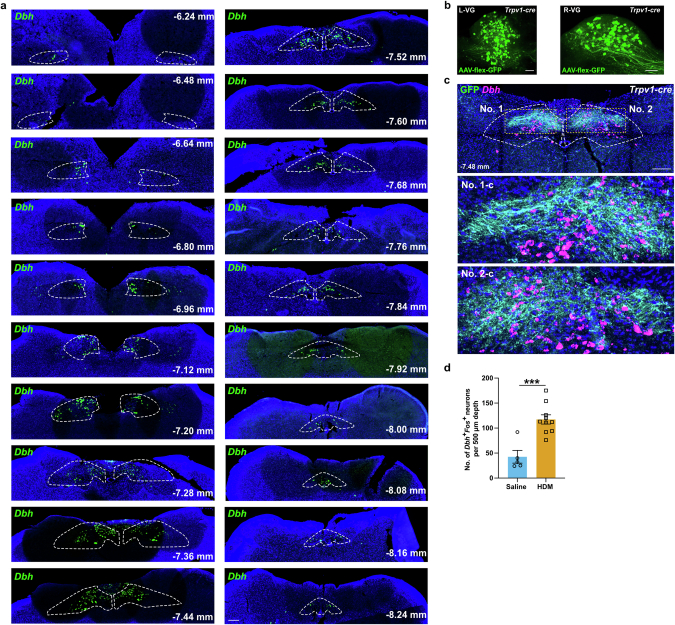
*Dbh*^*+*^ neurons in the nTS were activated upon allergen challenge to lung. (**a**) RNAscope of *Dbh* on serial sections (each section was at 20 µm thickness, 80 µm interval between sections) of naïve brainstem shows *Dbh* cover most of the nTS from Bregma −6.96 mm to Bregma −8.08 mm. *Dbh* is detected in an average of 2,342 ± 169 neurons in the nTS (*n* = 7 mice). (**b**) Vagal ganglia of *Trpv1*-cre mouse after bilateral microinjection of AAV-flex-GFP to the vagal ganglia. *n* = 6 mice. (**c**) GFP^+^ *Trpv1*^*+*^ vagal fibers project to the vicinity of *Dbh*^*+*^ neurons in the nTS. Boxed areas magnified at the bottom. *n* = 6 mice. (**d**) Quantification of the number of *Dbh*^*+*^*Fos*^*+*^ neurons in the nTS after HDM challenge to lung by RNAscope. Data are mean values ± s.e.m. *n* = 5 saline and 10 HDM mice, unpaired *t*-test (two-sided), *P* = 0.0004. Dashed circles in **a** and **c** outline nTS at indicated Bregma levels. Scale bars, 200 µm. Source Data

Compared with naive nTS, the same 18 neuron subtypes were detected in saline- and HDM-challenged nTS (Fig. 3a,b). To quantitatively determine the identity of allergen-activated nTS neurons, we performed double RNAscope of *Fos* with the top markers of each of the 18 nTS clusters. *Dbh*^*+*^ cluster 13 neurons showed the highest percentage of overlap with *Fos*^*+*^ neurons among all clusters assayed (Fig. 3c–e and Supplementary Table 3). Using RNAscope, *Dbh* was detected in an average of 2,342 ± 169 neurons in the nTS (Extended Data Fig. 5a). *Dbh*^*+*^ neurons reside in close proximity to *Trpv1*^*+*^ vagal projections, which are required for allergen-induced hyperreactivity^2^ (Extended Data Fig. 5b,c). Furthermore, the *Dbh*^*+*^ domain between bregma −6.96 and −8.08 mm overlaps substantially with that where allergen-induced FOS^+^ cells were enriched (bregma −7.20 to −8.08 mm; Extended Data Fig. 1k–v). Compared with saline, *Fos*^*+*^*Dbh*^*+*^ nTS neurons were increased following HDM challenge (Extended Data Fig. 5d). Together, these data led us to focus on *Dbh*^*+*^ nTS neurons to determine their role in allergen responses.

## *Dbh*^+^ neurons are necessary for hyperreactivity

**Fig. 4:**
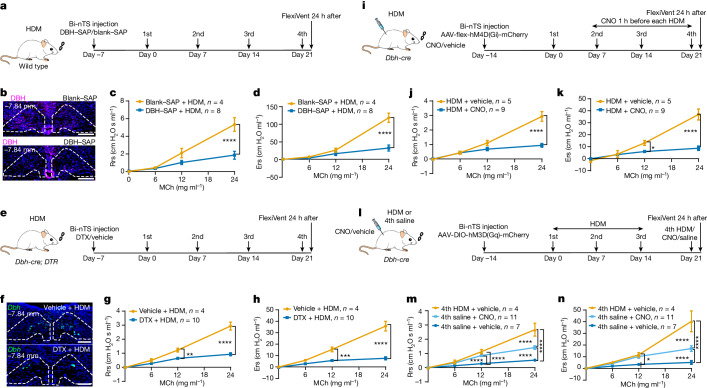
*Dbh*^*+*^ neurons in the nTS mediate airway hyperreactivity. **a**, Experimental scheme for chemical ablation of *Dbh*^*+*^ nTS neurons. Bi-nTS, bilateral nTS. **b**, DBH antibody staining in nTS. **c**,**d**, FlexiVent data showing blunted airway hyperreactivity following DBH–SAP treatment. *n* = 4 blank–SAP and *n* = 8 DBH–SAP mice, unpaired *t*-test at 0, 6, 12 and 24 mg ml^−1^ MCh separately, at 24 mg ml^−1^ MCh of both Rrs (**c**) and Ers (**d**), *P* < 0.0001; NS for all other pairwise comparisons. **e**, Experimental scheme for genetic ablation of *Dbh*^*+*^ nTS neurons. **f**, *Dbh* RNAscope in nTS. **g**,**h**, FlexiVent data showing blunted airway hyperreactivity following DTX injection. *n* = 4 vehicle and *n* = 10 DTX mice, unpaired *t*-test, for Rrs (**g**) at 12 mg ml^−1^ MCh, *P* = 0.0022; for Ers (**h**) at 12 mg ml^−1^ MCh, *P* = 0.0002; and for both Rrs (**g**) and Ers (**h**) at 24 mg ml^−1^, *P* < 0.0001; NS for all other pairwise comparisons. **i**, Experimental scheme for chemogenetic inhibition of *Dbh*^*+*^ nTS neurons. **j**,**k**, FlexiVent data showing blunted airway hyperreactivity following CNO injection. *n* = 5 vehicle and *n* = 9 CNO mice, unpaired *t*-test, for Rrs (**j**) at 24 mg ml^−1^ MCh, *P* < 0.0001; for Ers (**k**) at 12 mg ml^−1^ MCh, *P* = 0.0370, at 24 mg ml^−1^ MCh, *P* < 0.0001; NS for all other pairwise comparisons. **l**, Experimental scheme for chemogenetic activation of *Dbh*^*+*^ nTS neurons. **m**,**n**, FlexiVent data showing partially increased airway hyperreactivity following CNO injection, in place of the fourth HDM. All groups received the first, second and third HDM challenges. *n* = 4 fourth HDM, *n* = 11 fourth CNO and *n* = 7 fourth saline mice, multiple-comparisons one-way ANOVA (Bonferroni post hoc test), for Rrs (**m**) at 12 mg ml^−1^ MCh, *P* < 0.0001 between fourth HDM and fourth saline, between fourth CNO and fourth saline, at 24 mg ml^−1^ MCh, *P* < 0.0001 for all pairs; for Ers (**n**) at 12 mg ml^−1^ MCh, *P* = 0.0384 between fourth HDM and fourth saline, *P* = 0.0252 between fourth CNO and fourth saline, at 24 mg ml^−1^ MCh, *P* < 0.0001 for all pairs; NS for all other pairwise comparisons. Data are mean ± s.e.m., two-sided for unpaired *t*-test. **b**,**f**, Scale bars, 200 µm. Source Data

**Extended Data Fig. 6:**
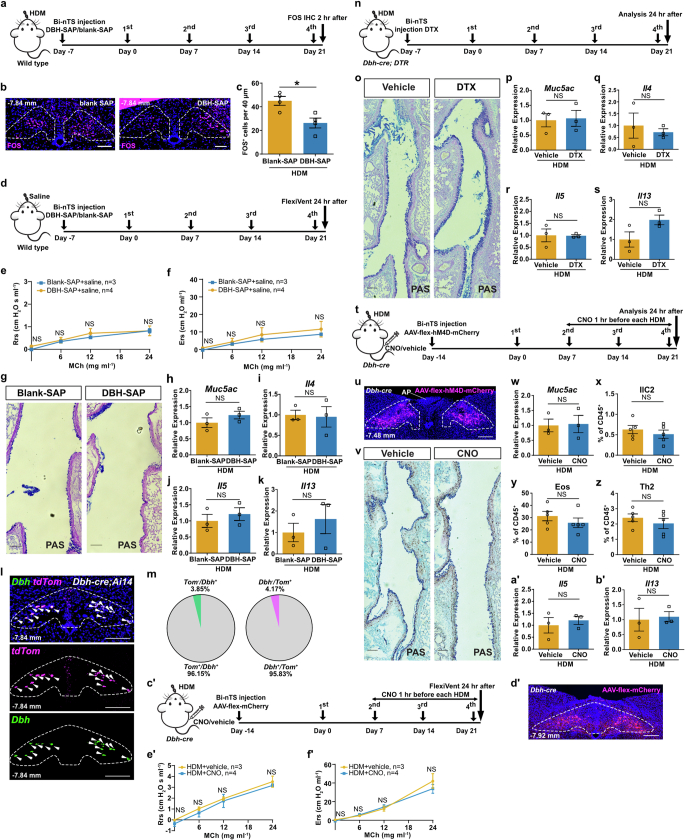
Chemical depletion, genetic depletion or genetic inactivation of *Dbh*^+^ neurons did not affect goblet cell metaplasia or cytokine gene expressions. (**a**) Experiment schemes for FOS^+^ cells detection after DBH-SAP injection and HDM. (**b**,**c**) Representative staining (**b**) and quantification (**c**, *n* = 4 blank-SAP and 4 DBH-SAP mice, unpaired *t*-test, *P* = 0.0155) showing reduced FOS^+^ cells following DBH-SAP treatment. (**d**) Experiment scheme for flexiVent after DBH-SAP injection and saline treatment. (**e**,**f**) FlexiVent, *n* = 3 blank-SAP and 4 DBH-SAP mice, unpaired *t*-test, NS. for all pairwise comparisons. (**g**-**k**) PAS staining (**g**) and qPCR (**h**-**k**) showing no change in goblet cell metaplasia or expression of type 2 cytokine genes. n = 3 blank-SAP and 3 DBH-SAP mice, unpaired *t*-test, NS. (**l**,**m**) Representative RNAscope (**l**) and quantification (**m**, *n* = 3 mice) showing extensive overlap of signals (arrowheads in **l**). (**n**) Experiment scheme for DTX-mediated ablation of *Dbh*^+^ neurons in the nTS. (**o**-**s**) PAS staining (**o**) and qPCR (**p**-**s**) showing that *Dbh*^*+*^ neuron ablation has no effect on goblet cell metaplasia or expression of type 2 cytokine genes. *n* = 3 vehicle and 3 DTX mice, unpaired *t*-test, NS. (**t**) Experiment scheme for *Dbh*^+^ neuron chemogenetic inhibition. (**u**) Representative AAV-flex-hM4D-mCherry signals in the nTS. (**v**,**w**) PAS staining (**v**) and qPCR (**w**, *n* = 3 vehicle and 3 CNO mice, unpaired *t*-test, NS) showing that chemogenetic inhibition of *Dbh*^*+*^ nTS neurons has no effect on goblet cell metaplasia. (**x**-**b**’) Flow cytometry analyses of ILC2s, Eos and Th2, and qPCR from whole lungs showing that chemogenetic inhibition of *Dbh*^*+*^ nTS neurons has no effect on immune cell infiltration. For **x**-**z**, *n* = 5 vehicle and 5 CNO mice, for **a**’ and **b**’, *n* = 3 vehicle and 3 CNO mice, unpaired *t*-test, NS. (**c**’) Experiment scheme for control virus AAV-flex-mCherry injection. (**d**’) Representative AAV-flex-mCherry signals in the nTS. (**e**’,**f**’) FlexiVent, *n* = 3 vehicle and 4 CNO mice, unpaired *t*-test, NS for all pairwise comparisons. Scale bars, 100 µm (**g**,**o**,**v**), 200 µm (**b**,**l**,**u**, **d**’). Data are mean values ± s.e.m., statistical tests are two-sided. Source Data

To determine whether *Dbh*^*+*^ nTS neurons are essential for hyperreactivity, we used three approaches. First, we performed chemical ablation by injecting anti-dopamine beta-hydroxylase (DBH) antibody conjugated to saporin (SAP), shown to be specific for DBH^+^ neurons^18^, into the nTS (Fig. 4a). Compared with scrambled, peptide-conjugated SAP (blank–SAP) control, the anti-DBH–SAP group showed a clear reduction in DBH^+^ neurons, confirming ablation efficiency (Fig. 4b). Following HDM treatment, the anti-DBH–SAP group showed reduced FOS^+^ cells compared with blank–SAP control (Extended Data Fig. 6a–c). In naive mice, DBH^+^ neuron ablation had no effect on methacholine responses (Extended Data Fig. 6d–f). In contrast, following HDM challenge, the anti-DBH–SAP-injected group showed blunted airway hyperreactivity compared with blank–SAP control (Fig. 4c,d).

Second, we performed genetic ablation. In *Dbh-cre; Ai14* mice, the RNA of *Dbh* and *tdTomato* showed over 95% overlap, confirming *cre* specificity (Extended Data Fig. 6l,m). We injected DTX into the nTS of *Dbh-cre*; *Rosa-lxl-DTR* (*Dbh-cre*; *DTR*) mice (Fig. 4e), leading to efficient loss of *Dbh*^+^ neurons in the nTS (Fig. 4f). Following ablation, we carried out HDM challenge and found that allergen-induced airway hyperreactivity was blunted in the DTX-injected group compared with the vehicle-injected control group (Fig. 4g, h).

Third, we performed chemogenetic inactivation using the designer receptors exclusively activated by designer drugs (DREADD) system. We injected AAV-flex-hM4D(Gi)-mCherry into the nTS of *Dbh-cre* mice, eliciting efficient and specific expression (Fig. 4i and Extended Data Fig. 6t,u). We administered clozapine-*N*-oxide (CNO) to activate hM4D(Gi) 1 h before the second to fourth HDM, sparing the first HDM which is required for sensitization of the immune system^2^. We found that the CNO group showed blunted airway hyperreactivity response compared with the vehicle group (Fig. 4j,k). In mice expressing mCherry without hM4D(Gi), administration of CNO had no effect (Extended Data Fig. 6c’–f’).

**Extended Data Fig. 7:**
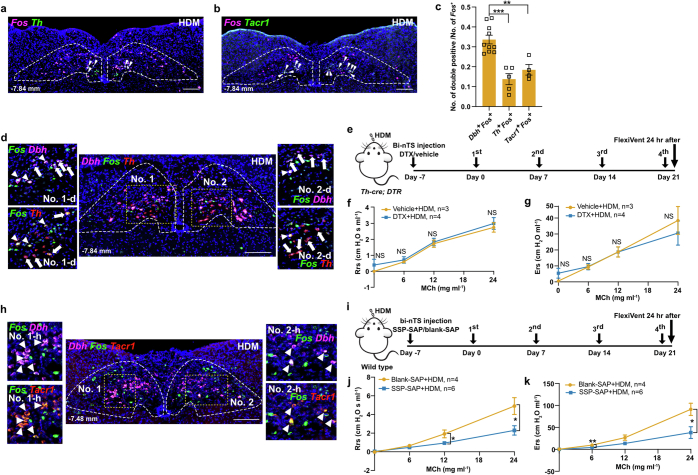
Ablation of *Tacr1*^*+*^ neurons, but not *Th*^*+*^ neurons in the nTS led to reduced airway hyperreactivity. (**a**,**b**) Double RNAscope showing the overlap between *Th* and *Fos* (**a**), *Tacr1* and *Fos* (**b**) in the nTS at 1.5 h after the fourth HDM challenge to lung. Arrowheads indicate cells with overlapping signals. (**c**) Quantification of *Th*^*+*^*Fos*^*+*^ (*n* = 5 mice) and *Tacr1*^*+*^*Fos*^*+*^ (*n* = 4 mice) neurons as compared to *Dbh*^*+*^*Fos*^*+*^ (*n* = 10 mice) neurons in the nTS after the fourth HDM challenge to lung by RNAscope. Multiple comparisons under one-way ANOVA (Bonferroni post hoc test), *P* = 0.0001 between *Dbh*^*+*^*Fos*^*+*^ and *Th*^*+*^*Fos*^*+*^, *P* = 0.0020 between *Dbh*^*+*^*Fos*^*+*^ and *Tacr1*^*+*^*Fos*^*+*^, NS between *Th*^*+*^*Fos*^*+*^ and *Tacr1*^*+*^*Fos*^*+*^. (**d**) Representative image showing *Dbh*^*+*^*Fos*^*+*^ neurons and *Th*^*+*^*Fos*^*+*^ neurons have only a few triple overlaps by RNAscope. Arrows indicate *Dbh*^*+*^*Fos*^*+*^ neurons or *Th*^*+*^*Fos*^*+*^ neurons, arrowheads indicate overlapping *Dbh*^*+*^*Th*^*+*^*Fos*^*+*^ neurons. (**e**) Experiment scheme for genetic ablation of *Th*^*+*^ nTS neurons. (**f**,**g**) FlexiVent data showing no effect on airway hyperreactivity after DTX injection. *n *= 3 vehicle and 4 DTX mice, unpaired *t*-test (two-sided), NS for all pairwise comparisons. (**h**) Representative image showing *Dbh*^*+*^*Fos*^*+*^ neurons and *Tacr1*^*+*^*Fos*^*+*^ neurons have clear triple overlaps by RNAscope. Arrowheads indicate overlapping *Dbh*^*+*^*Tacr1*^*+*^*Fos*^*+*^ neurons. (**i**) Experiment scheme for chemical ablation of *Tacr1*^*+*^ nTS neurons. (**j**,**k**) FlexiVent data showing blunted airway hyperreactivity after SSP-SAP treatment. *n* = 4 blank-SAP and 6 SSP-SAP mice, unpaired *t*-test (two-sided), for Rrs (**j**), at 12 mg ml^−1^ MCh, *P* = 0.0224, at 24 mg ml^−1^, *P* = 0.0254; for Ers (**k**), at 6 mg ml^−1^, *P* = 0.0033, at 24 mg ml^−1^, *P* = 0.0297. NS for all other pairwise comparisons. Outlines in **a**,**b**,**d** and **h** delineate nTS regions at indicated Bregma levels. Boxed areas in **d** and **h** are magnified as labeled. Scale bars, 200 µm. Data are mean values ± s.e.m. Source Data

We also tested other nTS populations for their requirement in the allergen circuit. Our early RNAscope data showed that both *Th*^19^ and *Tacr1* (ref. ^20^) had some overlap with *Fos* (Extended Data Fig. 7a–c). Furthermore, there is minimal overlap between *Th*^*+*^*Fos*^*+*^ neurons and *Dbh*^*+*^*Fos*^*+*^ neurons (Extended Data Fig. 7d). Ablation of *Th*^*+*^ nTS neurons by DTX into the nTS of *Th-cre*; *Rosa-lxl-DTR* mice did not affect allergen-induced airway hyperreactivity (Extended Data Fig. 7e–g). In contrast, most, if not all, *Tacr1*^*+*^*Fos*^*+*^ neurons are also *Dbh*^*+*^ (Extended Data Fig. 7h). We found that chemical ablation of *Tacr1*^*+*^ nTS neurons using anti-substance P receptor (SSP/TACR1) antibody conjugated to saporin (SSP–SAP) blunted hyperreactivity (Extended Data Fig. 7i–k). Together, the data from chemical ablation, genetic ablation and chemogenetic inactivation demonstrate that *Dbh*^*+*^ nTS neurons are necessary for allergen-induced airway hyperreactivity.

## *Dbh*^*+*^ neurons can induce hyperreactivity

**Extended Data Fig. 8:**
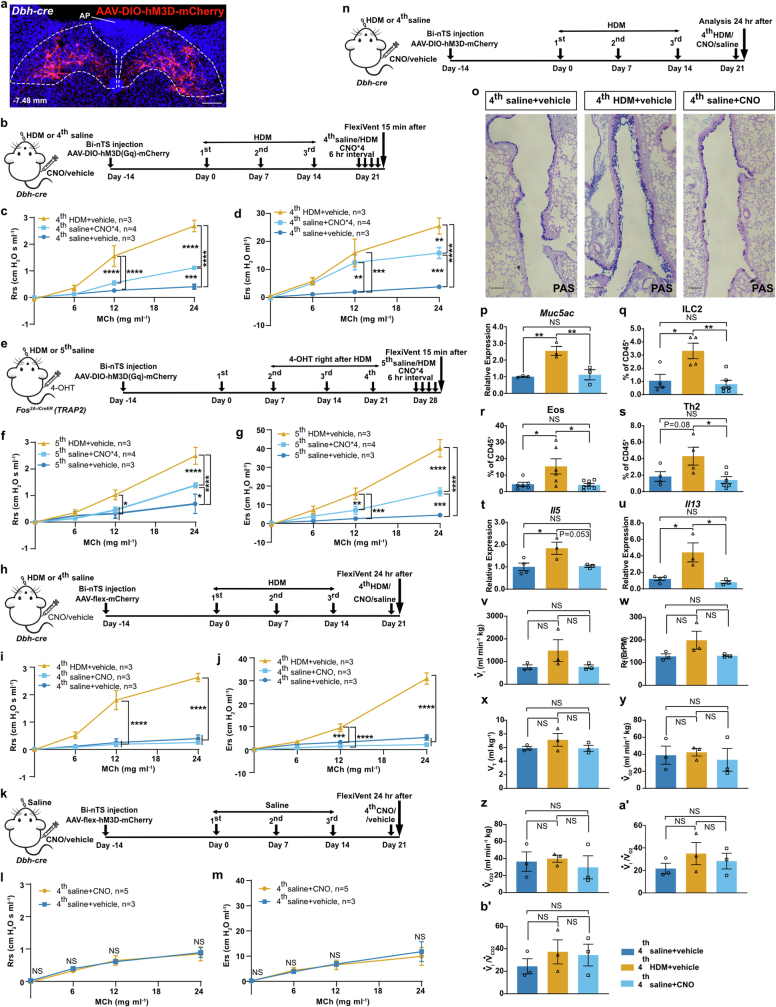
Chemogenetic activation of *Dbh*^+^ nTS neurons did not affect goblet cell metaplasia, immune cell infiltration, or respiratory parameters. (**a**) Representative mCherry signals (with Dsred antibody staining) following AAV-DIO-hM3D-mCherry injection in the nTS (outlined, no expression in AP). (**b**) Experiment scheme for repeated chemogenetic activation of *Dbh*^+^ nTS neurons. (**c**,**d**) FlexiVent, *n* = 3 HDM, *n* = 4 CNO*4 and *n* = 3 Saline mice, multiple comparisons under one-way ANOVA (Bonferroni post hoc test), for Rrs, at 12 and 24 mg ml^−1^ MCh, *P* < 0.0001 HDM versus saline or CNO*4, at 24 mg ml^−1^, *P* = 0.0010 CNO*4 versus saline, *P* < 0.0001 for the other two pairs; for Ers, at 12 mg ml^−1^, *P* = 0.0016 CNO*4 versus saline, *P* = 0.0001 HDM versus saline; at 24 mg ml^−1^, *P* = 0.0003 CNO*4 versus saline, *P* < 0.0001 HDM versus saline, *P* = 0.0034 HDM versus CNO*4. NS for remaining pairs. (**e**) Experiment scheme for repeated chemogenetic activation of allergen-activated *Fos2A-iCreER* (*TRAP2*) neurons. (**f**,**g**) FlexiVent, *n* = 3 HDM, *n* = 4 CNO*4 and *n* = 3 saline mice, multiple comparisons one-way ANOVA (Bonferroni post hoc test), for Rrs, at 12 mg ml^−1^ MCh, *P* = 0.0162 HDM versus saline, *P* = 0.0392 HDM versus CNO*4, at 24 mg ml^−1^, *P* = 0.0108 CNO*4 versus saline, *P* < 0.0001 for the other two pairs; for Ers, at 12 mg ml^−1^, *P* = 0.0002 HDM versus saline, *P* = 0.0074 HDM versus CNO*4, at 24 mg ml^−1^, *P* = 0.0002 CNO*4 versus saline, *P* < 0.0001 for the other two pairs. NS for remaining pairs. (**h**) Experiment scheme for control virus AAV-flex-mCherry injection to the nTS of *Dbh-cre* mice. (**i**,**j**) FlexiVent, *n* = 3 HDM, *n* = 3 saline and *n* = 3 CNO mice, multiple comparisons one-way ANOVA (Bonferroni post hoc test), for Rrs (**i**), at 12 and 24 mg ml^−1^ MCh, *P* < 0.0001 HDM versus saline or CNO; for Ers (**j**), at 12 mg ml^−1^, *P* = 0.0004 HDM versus saline, *P* < 0.0001 HDM versus CNO, at 24 mg ml^−1^, *P* < 0.0001 HDM versus saline or CNO. NS for remaining pairs. (**k**) Experiment scheme for activation of nTS *Dbh*^+^ neurons in naïve mice. (**l**,**m**) FlexiVent, *n* = 5 CNO versus *n* = 3 vehicle mice, unpaired *t*-test (two-sided), NS. (**n**) Experiment scheme for activation of nTS *Dbh*^+^ neurons in sensitized mice. (**o**,**p**) PAS staining (**o**) and qPCR (**p**). *n* = 3 mice for each group, unpaired *t*-test (two-sided), *P* = 0.0058 HDM versus saline, *P* = 0.0078 HDM versus CNO; NS CNO versus saline. (**q**-**u**) Flow cytometry (**q** and **s**, *n* = 4 saline, *n* = 4 HDM and *n* = 6 CNO; **r**, *n* = 6 saline, *n* = 6 HDM and *n* = 8 CNO) and qPCR (**t**,**u**, *n* = 4 saline, *n* = 3 HDM and *n* = 3 CNO). Multiple comparisons under one-way ANOVA (Bonferroni post hoc test), for **q**, *P* = 0.0144 HDM versus saline, *P* = 0.0040 HDM versus CNO; for **r**, *P* = 0.0261 HDM versus saline, *P* = 0.0141 HDM versus CNO; for **s**, *P* = 0.0256 HDM versus CNO; for** t**, *P* = 0.0344 HDM versus saline; for **u**, *P* = 0.0169 HDM versus saline, *P* = 0.0131 HDM versus CNO. NS for remaining pairs. (**v**-**b**’) Measurements of respiratory parameters. *n* = 3 mice for each group, multiple comparisons under one-way ANOVA (Bonferroni post hoc test), NS. Scale bars, 200 µm (**a**), 100 µm (**o**). Data are mean values ± s.e.m. Source Data

To address whether *Dbh*^*+*^ neurons are sufficient for driving allergen response, we used DREADD-targeted activation. We injected AAV-DIO-hM3D(Gq)-mCherry into the nTS of *Dbh-cre* mice (Fig. 4l and Extended Data Fig. 8a), then administrated CNO in place of the fourth HDM challenge. Although not to the full extent as HDM, CNO activation was partially sufficient to induce increased airway hyperreactivity compared with saline control (Fig. 4m,n). Partial hyperreactivity was also observed following repeated CNO-mediated activation of nTS *Dbh*^*+*^ neurons (Extended Data Fig. 8b–d) and following repeated CNO-mediated activation of allergen-induced *TRAP2* neurons in the nTS (Extended Data Fig. 8e–g). Injection of AAV-DIO-mCherry control virus did not induce airway hyperreactivity in sensitized airway (Extended Data Fig. 8h–j). In naive mice not exposed to HDM, CNO activation (the fourth dose) of *Dbh*^*+*^ neurons had no effect (Extended Data Fig. 8k–m).

In none of the three loss-of-function experiments, nor in the chemogenetic gain-of-function experiment or *TRAP2; Ai14; DTR* experiments, did we observe any changes in HDM-induced goblet cell metaplasia, immune cell infiltration or expression of *Il4*, *Il5* and *Il13* (Extended Data Figs. 3p–x, 6g–k,n–b’ and 8n–u). We also assayed for potential effects on other aspects of lung function. Following either HDM challenge or CNO activation, compared with saline controls, we found no statistically significant difference in minute ventilation, respiratory frequency, tidal volume or metabolic rate as measured by plethysmography (Extended Data Fig. 8v–b’). This is consistent with a previous report that HDM challenge in mice did not alter respiratory parameters^21^. These results indicate that CNO activation of *Dbh*^*+*^ nTS neurons induced airway hyperreactivity in sensitized airways without affecting respiration. Together, data from functional tests demonstrate that *Dbh*^*+*^ nTS neurons are necessary and partially sufficient for allergen-induced airway hyperreactivity.

## Downstream NA and parasympathetic neurons

**Fig. 5:**
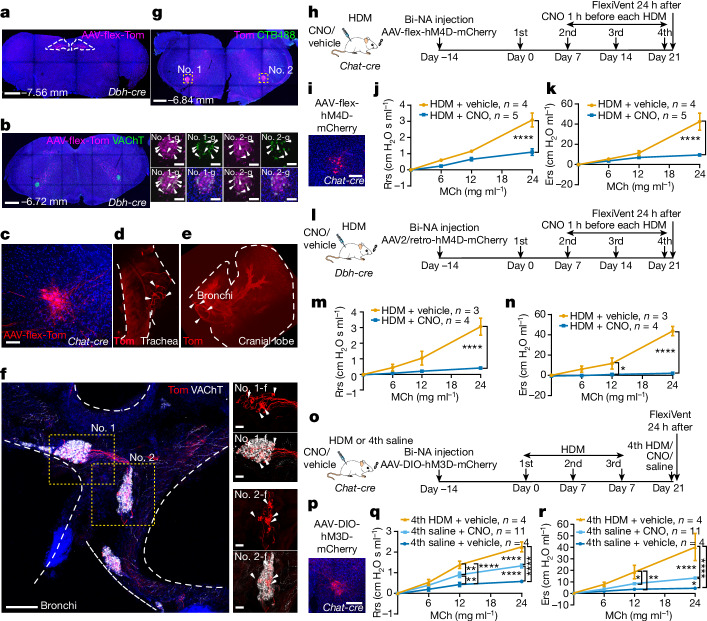
Parasympathetic neurons in the NA are necessary and sufficient downstream of the nTS for allergen-induced airway hyperreactivity. **a**, Brainstem section showing injection of AAV-flex-tdTomato into *Dbh-cre* mouse. **b**, In the same mouse, tdTom^+^ nerves project to the NA (vesicular acetylcholine transporter; VAChT^+^). **c**, Injection of AAV-flex-tdTomato into the NA of a *Chat-cre* mouse. **d**,**e**, In the same mouse, tdTom^+^ projects to both trachea (**d**) and extrapulmonary bronchi (**e**). **f**, NA-originated tdTom^+^ fibres innervate postganglionic parasympathetic ganglia (VAChT^+^) on extrapulmonary bronchi. Arrowheads indicate innervated signals. **g**, In *Chat-cre; Ai14* mice, injection of CTB488 in dorsal trachea labelled CTB488^+^tdTom^+^ NA neurons. Arrowheads indicate overlapping expression. **h**, Scheme for chemogenetic inhibition of *Chat*^+^ neurons in bilateral NA (bi-NA). **i**, AAV-flex-hM4D-mCherry signals. **j**,**k**, FlexiVent, *n* = 4 vehicle and *n* = 5 CNO mice, unpaired *t*-test, at 24 mg ml^−1^ MCh, *P* < 0.0001 for both Rrs (**j**) and Ers (**k**); NS for all other pairs. **l**, Scheme for inhibiting NA-innervating *Dbh*^+^ nTS neurons by injecting AAV2/retro-flex-hM4D-mCherry bilaterally into NA of *Dbh-cre* mice. **m**,**n**, FlexiVent, *n* = 3 vehicle and *n* = 4 CNO mice, unpaired *t*-test, for Ers (**n**), at 12 mg ml^−1^ MCh, *P* = 0.0127; at 24 mg ml^−1^ MCh, *P* < 0.0001 for both Rrs (**m**) and Ers (**n**); NS for all other pairs. **o**, Scheme for chemogenetic activation of *Chat*^+^ neurons in bilateral NA. **p**, AAV-DIO-hM3D-mCherry signals. **q**,**r**, FlexiVent, *n* = 4 fourth HDM, *n* = 11 fourth CNO and *n* = 4 fourth saline mice, multiple-comparisons one-way ANOVA (Bonferroni post hoc test), for Rrs (**q**), at 12 mg ml^−1^ MCh, *P* < 0.0001 between fourth HDM and fourth saline, *P* = 0.0092 between fourth CNO and fourth saline, *P* = 0.0081 between fourth HDM and fourth CNO; at 24 mg ml^−1^ MCh, *P* < 0.0001 for all comparisons; for Ers (**r**), at 12 mg ml^−1^ MCh, *P* = 0.0022 between fourth HDM and fourth saline, *P* = 0.0145 between fourth HDM and fourth CNO; at 24 mg ml^−1^ MCh, *P* = 0.0371 between fourth CNO and fourth saline, *P* < 0.0001 for remaining pairs; NS for all other pairwise comparisons. Data are mean ± s.e.m., two-sided for unpaired *t*-test. Scale bars, 500 µm (**a**,**b**), 100 µm (**c**,**i**,**p**), 200 µm (**f**, 50 µm in magnified views; **g**, 100 µm in magnified views). Source Data

**Extended Data Fig. 9:**
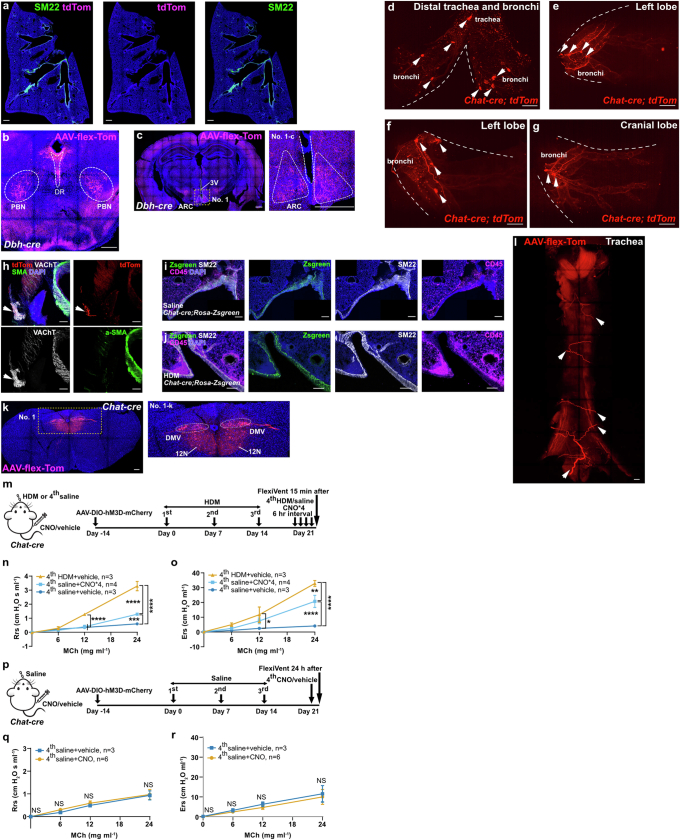
Parasympathetic neurons in the NA project to the airway-innervating postganglionic neurons. (**a**-**c**) tdTom^+^ fibers from Fig. 5a were absent in the lung (**a**), while present in the lateral parabrachial nucleus (PBN, **b**) and dorsal raphe nucleus (DR, **b**) and arcuate hypothalamic nucleus (ARC, **c**). 3 V, the third ventricle. Yellow boxed areas are magnified on the right. (**d**-**g**) tdTom^+^ neurons/ganglia (arrowheads) reside in the trachea, main bronchi (**d**) and main airway entrance (hilum) of lung lobes (**e**-**g**) of *Chat-cre; tdTomato* mice. (**h**) tdTom^+^ nerves innervate postganglionic parasympathetic ganglia (VAChT^+^, arrowhead) in the extrapulmonary airway, rather than directly on smooth muscle cells (SMA^+^). (**i**,**j**) Zsgreen^+^ parasympathetic nerve terminals near both airway smooth muscles and immune cells. (**k**) Stereotaxic injection of AAV-flex-tdTomato into bilateral DMV (also labeled neurons in ventral adjoining 12 N) of *Chat-cre* mice. Yellow boxed area was magnified on the right, ovals outline DMV regions. (**l**) tdTom^+^ nerves were found passing through the space between trachea and esophagus (arrowheads). *n* = 3 mice for **a**-**l**. (**m**) Experiment scheme for measuring airway hyperreactivity after repeated chemogenetic activation of *Chat*^+^ neurons in the NA. (**n**,**o**) FlexiVent, *n* = 3 fourth HDM, 4 fourth CNO*4 and 3 fourth saline mice, multiple comparisons under one-way ANOVA (Bonferroni post hoc test), for Rrs (**n**), at 12 mg ml^−1^ MCh, *P* < 0.0001 fourth HDM versus fourth saline or fourth CNO, at 24 mg ml^−1^, *P* = 0.0002 fourth CNO*4 versus fourth saline, *P* < 0.0001 for remaining pairs; for Ers (**o**), at 12 mg ml^−1^, *P* = 0.0238 fourth HDM versus fourth saline, at 24 mg ml^−1^, *P* = 0.0014 fourth HDM versus fourth CNO*4, *P* < 0.0001 for remaining pairs. NS for all other pairs. (**p**) Experiment scheme for CNO activation of NA *Chat*^+^ neurons in naïve mice. Both groups received the 1st–3rd saline challenge. (**q**,**r**) FlexiVent, *n* = 3 fourth saline and 6 fourth CNO mice, unpaired *t*-test (two-sided), NS for all pairs. Scale bars, 500 µm (**a**,**d**-**g**), 200 µm (**b**-**c**,**i**,**j**,**k**,**l**), 100 µm (**h**). Data are mean values ± s.e.m. Source Data

To map *Dbh*^*+*^ nTS targets and their role in hyperreactivity, we injected AAV-flex-tdTomato into the nTS of *Dbh-cre* mice (Fig. 5a). No fibre was detected directly in the lung (Extended Data Fig. 9a). Following screening of the whole brainstem, we found tdTom^+^ fibres projecting to the NA (Fig. 5b). We also found fibres in the lateral parabrachial nucleus, dorsal raphe nucleus in the brainstem, arcuate hypothalamic nucleus and other hypothalamic structures, consistent with published data^22,23^ (Extended Data Fig. 9b,c).

To address whether cholinergic NA neurons project to the lung, we injected AAV-flex-tdTomato into the NA of *Chat-cre* mice (Fig. 5c). NA-originated tdTom^+^ fibres project to postganglionic parasympathetic ganglia residing in both the trachea and extrapulmonary bronchi, but not in the lung (Fig. 5d–f and Extended Data Fig. 9d–h). In turn, these postganglionic parasympathetic ganglia neurons project into the lung (Extended Data Fig. 9i,j). In comparison with NA, stereotaxic injection of AAV-flex-Tom into DMV and the adjacent 12N of *Chat-cre* mice labelled fibres that passed by the space between the trachea and oesophagus without innervating the trachea or bronchi (Extended Data Fig. 9k,l). To validate from the retrograde direction, we introduced CTB488 dorsal to the trachea, in which postganglionic parasympathetic ganglia are enriched. In whole-brainstem sections, we found cell body labelling in the NA, with little labelling in other regions (Fig. 5g).

To address NA function in hyperreactivity, we injected AAV-flex-hM4D(Gi)-mCherry into the NA of *Chat-cre* mice followed by the HDM regimen (Fig. 5h,i). The CNO group showed blunted airway resistance and elastance compared with vehicle-injected controls (Fig. 5j,k). To further validate the functional hierarchy between *Dbh*^*+*^ nTS neurons and NA neurons, we inhibited NA-innervating *Dbh*^*+*^ nTS neurons by injecting AAV2/retro-flex-hM4D-mCherry into the NA of *Dbh-cre* mice. Such inhibition was sufficient to abolish airway hyperreactivity (Fig. 5l–n).

To activate *Chat*^*+*^ neurons in the NA, we injected AAV-DIO-hM3D(Gq)-mCherry bilaterally into the NA of *Chat-cre* mice (Fig. 5o,p). Although not as potent as HDM, activation of *Chat*^*+*^ NA neurons by either single or repeated CNO injections was partially sufficient to induce increased airway hyperreactivity compared with saline (Fig. 5q,r and Extended Data Fig. 9m–o). In naive mice not exposed to HDM, CNO activation of *Chat*^*+*^ NA neurons had no effect (Extended Data Fig. 9p–r).

**Extended Data Fig. 10:**
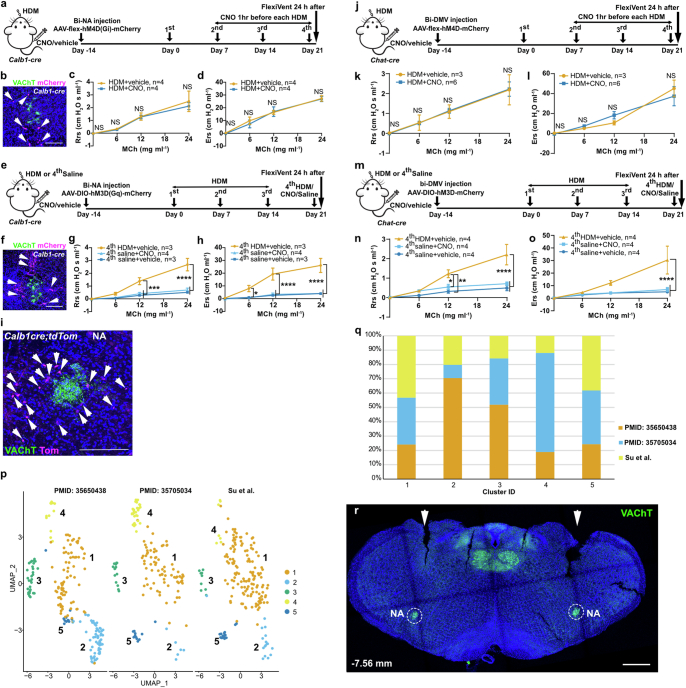
Modulation of *Calb1*^*+*^ neurons or parasympathetic neurons in the DMV did not affect allergen-induced airway hyperreactivity. (**a**) Experiment scheme for chemogenetic inhibition of *Calb1*^+^ neurons. Bi-DMV, bilateral DMV. (**b**) AAV-flex-hM4D-mCherry signals in the NA of *Calb1-cre* mouse. VAChT stains for cholinergic neurons in the NA. (**c**,**d**) FlexiVent, *n* = 4 HDM and 4 CNO mice, unpaired *t*-test (two-sided), NS for all pairs. (**e**) Experiment scheme for chemogenetic activation of *Calb1*^+^ neurons. (**f**) AAV-DIO-hM3D-mCherry signals in the NA of *Calb1-cre* mouse. VAChT stains for cholinergic neurons in the NA. (**g**,**h**) FlexiVent, *n* = 3 fourth HDM, 4 fourth CNO and 3 fourth saline mice, multiple comparisons under one-way ANOVA (Bonferroni post hoc test), for Rrs (**g**), at 12 mg ml^−1^ MCh, *P* = 0.0002 fourth HDM versus fourth saline, *P* = 0.0004 fourth HDM versus fourth CNO, at 24 mg ml^−1^, *P* < 0.0001 fourth HDM versus fourth saline or fourth CNO; for Ers (**h**), at 6 mg ml^−1^, *P* = 0.0418 fourth HDM versus fourth CNO, at 12 and 24 mg ml^−1^, *P* < 0.0001 fourth HDM versus fourth saline or fourth CNO. NS for all other pairs. (**i**) *Calb1-cre; tdTom* labeled neurons. (**j**) Experiment scheme for chemogenetic inhibition of *Chat*^+^ neurons in the DMV. (**k**,**l**) FlexiVent, *n* = 3 HDM and 6 CNO mice, unpaired *t*-test (two-sided), NS for all pairs. (**m**) Experiment scheme for chemogenetic activation of *Chat*^+^ neurons in the DMV. (**n**,**o**) FlexiVent, *n* = 4 fourth HDM, 4 fourth CNO and 4 fourth saline mice, multiple comparisons under one-way ANOVA (Bonferroni post hoc test), for Rrs (**n**), at 12 mg ml^−1^ MCh, *P* = 0.0023 fourth HDM versus fourth saline, *P* = 0.0248 fourth HDM versus fourth CNO, at 24 mg ml^−1^, *P* < 0.0001 fourth HDM versus fourth saline or fourth CNO; for Ers (**o**), at 24 mg ml^−1^, *P* < 0.0001 fourth HDM versus fourth saline or fourth CNO. NS for all other pairs. (**p**) UMAP plots of the integrated NA dataset. (**q**) Stacked bar plots. (**r**) Representative cryosection of the brainstem showing bilateral cannula implantation (tract indicated by arrowheads) target NA region (VAChT^+^). Arrowheads in **b**,**f**,**i**, labeled neurons that are outside of the region of the VAChT^*+*^ cholinergic NA neurons. Scale bars, 200 µm (**b**, **f**,**i**), 500 µm(**r**). Data are mean values ± s.e.m. Source Data

*Calb1*^*+*^ neurons in the general vicinity of the NA were shown to have a role in acute bronchoconstriction^12^. Using the same *Calb1-cre* as in ref. ^12^, modulation of *Calb1*^*+*^ neurons did not affect allergen-induced airway hyperreactivity (Extended Data Fig. 10a–h). On closer review, the results in the published study^12^ show that the *Calb1-cre* labelled neurons were in the AmbEx region, distant from the *Chat*^+^ NA region. Consistently, we found that *Calb1-cre*-labelled neurons are outside of the region in which VAChT^+^ NA neurons reside (Extended Data Fig. 10i). Furthermore, our DREADD-targeted neurons in *Calb1-cre* mice were located in regions surrounding VAChT^+^ cholinergic NA neurons but did not overlap with them (Extended Data Fig. 10b,f). Together, these data suggest that hyperreactivity is dependent on *Chat*^*+*^ NA neurons but not on nearby *Calb1*^*+*^ neurons.

To test whether DMV, a known target of nTS neurons^24^, may also act downstream in the allergen circuit, we performed DREADD-based inactivation or activation of *Chat*^*+*^ neurons in the DMV (Extended Data Fig. 10j,m). These DMV perturbations showed little effect on hyperreactivity (Extended Data Fig. 10k,l,n,o). Our data together demonstrate that NA neurons are necessary and partially sufficient for allergen-induced hyperreactivity.

## Noradrenaline to NA in hyperreactivity

**Fig. 6:**
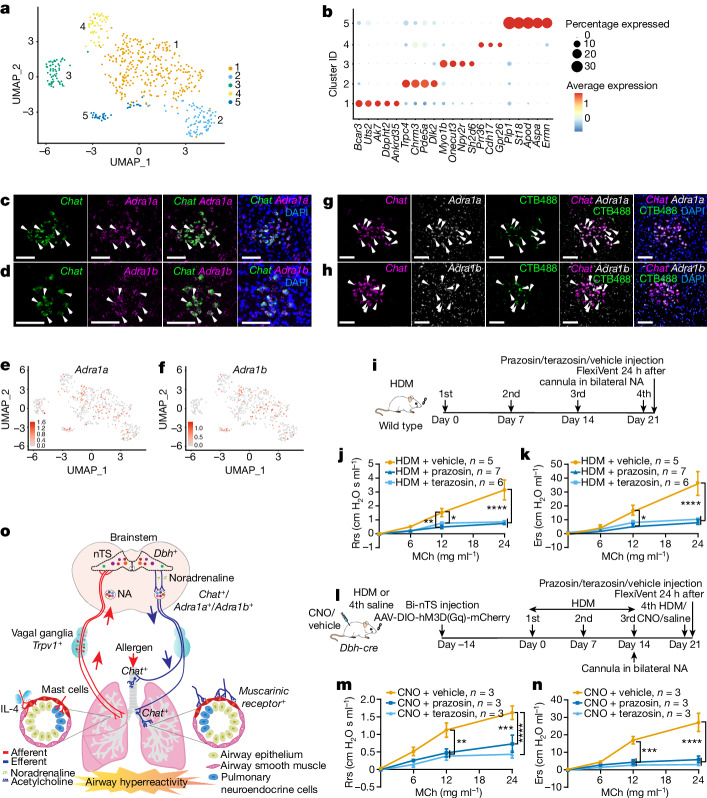
Blocking noradrenaline receptors in the NA blunted allergen-induced airway hyperreactivity. **a**, UMAP plot of our snRNA-seq data integrated with published datasets^12,13^. **b**, Dotplot showing top marker genes from the integrated dataset which overlap with those in published datasets^12,13^. Using default Seurat^29^ dotplot settings (Methods), percentage expressed was plotted from 0 to 30% detected, and the colour bar shows the average of the scaled normalized expression values across cells in a given cluster. **c**,**d**, Double RNAscope of NA showing overlap (arrowheads) for *Adra1a* (**c**) and *Adra1b* (**d**). **e**,**f**, Feature plots of *Adra1a* (**e**) and *Adra1b* (**f**). **g**,**h**, Injection of CTB488 into dorsal trachea in wild-type mouse labelled NA neurons for *Adra1a* (**g**) and *Adra1b* (**h**). **i**, Experimental scheme for noradrenaline receptor antagonist treatment. **j**,**k**, FlexiVent, *n* = 5 vehicle, *n* = 7 prazosin and *n* = 6 terazosin mice, multiple-comparisons one-way ANOVA (Bonferroni post hoc test), for Rrs (**j**), at 12 mg ml^−1^ MCh, *P* = 0.0017 prazosin versus vehicle, *P* = 0.0348 terazosin versus vehicle; at 24 mg ml^−1^ MCh, *P* < 0.0001 prazosin or terazosin versus vehicle; for Ers (**k**), at 12 mg ml^−1^ MCh, *P* = 0.0288 prazosin versus vehicle; at 24 mg ml^−1^ MCh, *P* < 0.0001 prazosin or terazosin versus vehicle; NS for all other pairwise comparisons. **l**, Experimental scheme for chemogenetic activation of the *Dbh*^+^ nTS neurons and delivering noradrenaline receptor antagonists into the NA of the same mouse. **m**,**n**, FlexiVent, *n* = 3 vehicle, *n* = 3 prazosin and *n* = 3 terazosin mice, multiple-comparisons one-way ANOVA (Bonferroni post hoc test), for Rrs (**m**), at 12 mg ml^−1^ MCh, *P* = 0.0047 prazosin versus vehicle, *P* = 0.0014 terazosin versus vehicle; at 24 mg ml^−1^ MCh, *P* = 0.0002 prazosin versus vehicle, *P* < 0.0001 terazosin versus vehicle; for Ers (**n**), at 12 mg ml^−1^ MCh, *P* = 0.0010 prazosin versus vehicle, *P* = 0.0002 terazosin versus vehicle; at 24 mg ml^−1^ MCh, *P* < 0.0001 prazosin or terazosin versus vehicle; NS for all other pairwise comparisons. **o**, Diagram illustrating multiple nodes of the complete allergen neural circuit. Data are mean ± s.e.m. Scale bars, 100 µm in **c**,**d**,**g**,**h**. Source Data

To determine the molecular nature of signalling from the nTS to NA, we profiled NA using snRNA-seq by dissection of the green fluorescent protein (GFP)^+^ region that corresponds to NA in *Chat-cre; CAG-Sun1/sfGFP* mice. Among all the neurons identified in the snRNA-seq dataset, we focused on the 188 *Chat*^*+*^ neurons. We then integrated our data with two recently published NA datasets^12,13^ following 2,000 unique feature counts cutoff. The integrated data with 534 NA neurons segregated into 5 clusters defined by markers that overlap with those published^12,13^ (Fig. 6a,b, Extended Data Fig. 10p,q and Supplementary Table 4).

Dopamine beta-hydroxylase converts dopamine to noradrenaline, raising the possibility that noradrenaline may be the signal between *Dbh*^*+*^ nTS and NA. Among all noradrenaline receptor genes, only *Adra1a* and *Adra1b* are expressed in the NA (Fig. 6c–f). Tracheal injection of CTB488 labelled *Adra1a*^*+*^ and *Adra1b*^*+*^ neurons in the NA (Fig. 6g,h). To address whether blocking of noradrenaline signal reception in the NA can blunt airway hyperreactivity, we infused either prazosin or terazosin, both of which are ADRA1 antagonists, into the NA (Fig. 6i and Extended Data Fig. 10r). Compared with vehicle control, targeted administration of prazosin or terazosin significantly blunted airway hyperreactivity (Fig. 6j,k). To address whether *Dbh*^*+*^ nTS neurons act through adrenergic signalling to the NA, we chemogenetically activated *Dbh*^*+*^ nTS neurons and delivered noradrenaline receptor antagonists into the NA of the same mouse (Fig. 6l). This blunted the ability of activated *Dbh*^*+*^ nTS neurons to trigger airway hyperreactivity (Fig. 6m,n). Together, these results suggest that noradrenaline acts as the neurotransmitter between *Dbh*^*+*^ nTS neurons and NA neurons in their function in allergen-induced airway hyperreactivity.

## Discussion

In this study, our findings delineate a full airway hyperreactivity neural circuit in which chronic allergen challenges to the lung are transmitted through immune cells and ascending vagal afferents to the *Dbh*^*+*^ nTS integrator, which then descend through efferent *Adra1a*^*+*^/*Adra1b*^*+*^ NA neurons before projecting back to postganglionic neurons and innervating the airway (Fig. 6o). This is a circuit identified in wild-type mice following repeated conditioning by allergen, suggesting the presence of an endogenous molecular and cellular response machinery. Repeated allergen exposure is central to asthma pathogenesis. Our findings defined a disease-relevant circuit with nodes from the immune system, the nervous system and structural cells of the organ.

We found that only repeated, but not single, allergen challenges led to a statistically significant increase in activated nTS neurons, similar to current-clamp recording data in the rhesus monkey^25^. Such chronic conditioning is distinct from mechanisms that drive acute airway constriction^26^. Our data demonstrate that ablation or inactivation of *Dbh*^*+*^ nTS neurons blunted the airway hyperreactivity present only following repeated allergen challenge. We note that this airway hyperreactivity circuit may utilize nodes that also have a role in acute airway constriction. For example, activation of thoracic cholinergic nerves in naive animals induced acute airway constriction in the absence of exogenous methacholine^26^. Our findings suggest that repeated allergen exposures may hijack this existing node to the delivery of elevated airway constriction above the threshold level of acute response, mimicking asthma. Our results further define the identity of the brainstem neurons that connect both afferent and efferent nodes to control airway hyperreactivity. The snRNA-seq datasets of both nTS and NA, together with existing lung and vagal neuron single-cell datasets, and the knowledge that acetylcholine is the probable neurotransmitter from NA to postganglionic neurons and then to airway smooth muscle cells^4,27,28^, can be used to complete the molecular connectome of the allergen circuit. Such molecular definition of the allergen-induced hyperreactivity circuit enables the use of neuromodulation to bypass the systemic side effects of current asthma treatments.

## Methods

### Mice

All mice were housed, and all experimental procedures were carried out in American Association for Accreditation of Laboratory, Animal Care-certified laboratory animal facilities at the University of California, San Diego (UCSD). All animal procedures were approved by the Institutional Animal Care and Use Committee at UCSD. Animals were maintained under constant environmental conditions (temperature in rooms is 68–72 °F and humidity 30–70%), with food and water provided ad libitum in a 12/12 h light/dark cycle. Adult mice from strains C57BL/6 J (no. JAX 000664), *c-Kit*^*w-sh/w-sh*^ (no. JAX 030764)*, Fos*^*2A-iCreER*^ (*TRAP2*, no. JAX 030323), *Rosa-lxl-tdTomato* (*Ai14*, no. JAX 007914), *Rosa-lxl-DTR* (no. JAX 016603), *Th*-cre (no. JAX 008601), *Chat-cre* (no. JAX 031661), *CAG-Sun1/sfGFP* (no. JAX 030952) and *Rosa-Zsgreen* (no. JAX 007906) were purchased from the Jackson laboratory. Strain *Dbh-cre* (MMRRC 036778) was purchased from the Mutant Mouse Resource and Research Center. All *cre* lines were maintained in a B6 background and were viable and fertile with no detectable abnormal phenotypes. Both male and female mice were used for experiments. Mice were at least 6 weeks old when subjected to HDM challenge, stereotaxic injection or surgery.

### HDM challenge

Mice were anaesthetized using isoflurane. 50 μg 20 μl^−1^ HDM extract (*Dermatophagoides pteronyssinus*, GREER Labs) was introduced intranasally for 4 consecutive weeks on days 0, 7, 14 and 21. For controls, 20 μl of saline was used instead of HDM on the same regime. Mice were euthanized 1.5 h following the last challenge for *Fos* RNAscope or nTS snRNA-seq; 2 h after the last challenge for FOS antibody immunostaining; 24 h after the last challenge for flexiVent assay, periodic acid–schiff (PAS) staining and quantitative PCR (qPCR); and 3 days after the last challenge for flow cytometry.

### Lung tissue PAS staining

Mice were euthanized by CO_2_ inhalation. The lungs were inflated with 4% paraformaldehyde (PFA) at 35 cm H_2_O airway pressure; they were postfixed in 4% PFA overnight and then prepared for paraffin sections at width 6 μm. Goblet cells were stained using a PAS staining kit (Sigma).

### Tissue collection and immunofluorescence staining

Mice were euthanized by CO_2_ inhalation followed by transcardial perfusion with PBS and 4% PFA. Subsequent to postfixing overnight in 4% PFA, tissues were washed in PBS followed by overnight sucrose dehydration. Brainstem blocks were sectioned at 25, 40 or 99 μm thickness in a rostral to caudal sequence, and lung blocks were sectioned at 99 or 300 μm thickness for parasympathetic neuron/airway staining. All sections were processed for immunostaining following the standard protocol. Primary antibodies used include rabbit anti-c-FOS (SYSY, no. 226 008, 1:300), rabbit anti-DBH (Sigma, no. AB1585, 1:300), rabbit anti-Dsred (Takara, no. 632496, 1:300), rabbit anti-VAChT (SYSY, no. 139 103, 1:300), mouse anti-alpha Smooth Muscle Actin-FITC (Sigma, no. F3777, 1:300), rabbit anti-TRPV1 (Alomone labs, no. ACC-030, 1:300) and chicken anti-GFP (abcam, no. ab13970, 1:300). Secondary antibodies used include goat anti-rabbit FITC, goat anti-rabbit Cy3 and goat anti-rabbit Cy5 (all from Jackson Immuno Research Labs, all 1:300). Slides were mounted with Vectashield (Vector Labs) and imaged using an Olympus VS200 Slide Scanner or Leica SP8 confocal microscope. For quantification of the total number of FOS^+^ neurons by antibody staining and the overlap between *Fos* and the top marker gene of each individual nTS cluster by RNAscope following HDM, we counted total cell numbers in 20 serial sections (at 25 µm thickness each, with 75 µm interval between sections, together representing ¼ of the whole nTS regions (2 mm)) and recorded these as one data point for one animal. For quantification of FOS^+^ neurons without the total label on the *y* axis, we counted three sections of nTS bregma regions with the most concentrated signals following serial section of the whole nTS (three sections were chosen between bregma −7.20 and −8.08 mm at the same stereotaxic coordinates between groups), and the average number was used as one data point for one animal. We used Qupath for overlap quantification in the nTS.

### RNAscope

Vagal ganglia or brainstems were sectioned at 25 μm thickness. All staining procedures were performed using the RNAscope Fluorescent Multiplex Kit (Advanced Cell Diagnostics, no. 320850) following the manufacturer’s instructions. The following probes from Advanced Cell Diagnostics were used: Mm-*Fos* (no. 316921), Mm-*Dbh* (no. 407851), Mm-*Phox2b* (no. 407861), Mm-*Otp* (no. 516391), Mm-*Mecom* (no. 432231), Mm-*Kcns3* (no. 467371), Mm-*Mafa* (no. 556931), Mm-*Egr3* (no. 431101), Mm-*Crym* (no. 466131), Mm-*Gbx2* (no. 314351), Mm-*Col23a1* (no. 432681), Mm-*Npy* (no. 313321), Mm-*Cdh3* (no. 514591), Mm-*Nr4a2* (no. 423351), Mm-*Lhx9* (no. 495431), Mm-*Pou3f1* (no. 436421), Mm-*Tac2* (no. 446391), Mm-*St18* (no. 443271), Mm-*Igf1* (no. 443901), Mm-*Lama2* (no. 424661), Mm-*Th* (no. 317621), Mm-*Tacr1* (no. 428781), Mm-*Trpv1* (no. 313331), Mm-*Gfp* (no. 400281), Mm-*tdTomato* (no. 317041), Mm-*Chat* (no. 408731), Mm-*Adra1a* (no. 408611) and Mm-*Adra1b* (no. 413561).

### Vagotomy

Mice were anaesthetized with a mixture of ketamine (100 mg kg^−1^) and xylazine (10 mg kg^−1^) by intraperitoneal injection (the same approach was used for all anaesthesia in our study unless otherwise notated). Fully anaesthetized mice were placed ventral side up on a stereotaxic frame, then 70% ethanol was sprayed on the throat to wet the fur. The skin was lifted to make a vertical cut (1 cm) on the throat, then one side of the vagal nerve was isolated and teased away from the carotid artery using small, curved forceps. Unilateral vagotomy was conducted by lifting the vagal nerve and cutting with straight scissors. Control sham operations were performed by lifting the vagal nerve and releasing it intact. Following vagotomy, the wounds were sutured and the area was disinfected with povidone-iodine. For all surgeries in our study, unless otherwise notated, mice were positioned on a heating pad to maintain body temperature and ophthalmic ointment was applied to maintain eye lubrication during surgery. Postoperative analgesia was provided with Buprenorphine SR (0.1 mg kg^−1^, subcutaneous injection). Mice were allowed to recover for 1 week before being subjected to allergen challenge.

### 4-OHT injection and *TRAP2* labelling

4-Hydroxytamoxifen (4-OHT, Sigma, no. H6278) was dissolved at 20 mg ml^−1^ in ethanol by shaking at 37 °C for 15 min, followed by aliquoting and storage at −20 °C for up to several weeks. Before use, 4-OHT was redissolved in a 1:4 mixture of castor oil/sunflower seed oil (Sigma, nos. 259853 and S5007). Ethanol was fully evaporated by vacuum under centrifugation. To determine brainstem neurons activated by allergen challenge but not by food consumption^30^, mice were fasted for 12 h before allergen challenge and 4-OHT injection; they were then placed back on a regular diet following *cre* activation. Mice were maintained in their home cages for 1 week to allow tdTomato expression before treatment.

### CLARITY-based brain clearing

A hydrogel based on 1% acrylamide (1% acrylamide, 0.125% Bis, 4% PFA, 0.025% (w/v) VA-044 initiator, in 1× PBS) was used for all CLARITY preparations. Following transcardial perfusion with 4% PFA and postfixation, brainstems were transferred to 1% hydrogel for 48 h to allow monomer diffusion. Samples were degassed and polymerized for 4–5 h at 37 °C. Samples were washed with 200 mM NaOH-boric buffer (pH 8.5) containing 8% SDS for 6–12 h and then transferred to a flow-assisted clearing device using a temperature-control circulator. Next, 100 mM Tris-boric buffer (pH 8.5) containing 8% SDS was used to accelerate clearing, after which samples were washed in PBS + 0.1% Triton X for at least 24 h at 37 °C. Samples were incubated in a refractive index-matching solution (refractive index = 1.45) for 8 h at 37 °C and then 6–8 h at room temperature before confocal imaging.

### Brainstem stereotaxic injection

Fully anaesthetized mice were placed in a stereotaxic frame with the head angled at 45°. A midline incision was made though the animal’s skin, posterior neck muscles and dura mater were pulled to expose the medulla between the occipital bone and C1 vertebra. Based on the stereotaxic coordinates of mouse brain^31^ and using obex as a reference point, injections were made into either bilateral nTS (0.1 mm rostral to obex, 0.2 mm lateral to midline, 0.25 mm under the medullary surface), bilateral NA (0.65 mm caudal to obex, 1.25 mm lateral to midline, 0.45 mm under the medullary surface) or bilateral DMV (0.05 mm caudal to obex, 0.1 mm lateral to midline, 0.1 mm under the medullary surface) using a calibrated glass micropipette attached to a Nanoject II injector (Drummond) and microprocessor pump (Pneumatic PicoPump, WPI). Each injection lasted no less than 10 min. Following injection, the glass micropipette was left in place for an additional 10 min before slow withdrawal. DTX (2 ng 200 nl^−1^), anti-DBH–SAP (42 ng 200 nl^−1^, advanced targeting system, no. IT-03), SSP–SAP (3.25 ng 200 nl^−1^, advanced targeting system, no. IT-11), blank–SAP (advanced targeting system, no. IT-21) or virus (AAV2/9-flex-tdTomato, 1.3 × 10^13^ genome copies (gc) ml^−1^, AAV2/8-flex-hM4D(Gi)-mCherry, 2.07 × 10^13 ^gc ml^−1^, AAV2/8-DIO-hM3D(Gq)-mCherry, 8 × 10^12 ^gc ml^−1^, AAV2/8-CMV-flex-TVA-mCherry-2A-oG, 1.39 × 10^13 ^gc ml^−1^, EnvA G-Deleted Rabies-EGFP, 5.0 × 10^7 ^gc ml^−1^; Boston Children’s Hospital Vector Core and Salk GT3 core) were used. Blank–SAP cannot enter cells and is thus non-toxic to neurons, serving as the appropriate control for DBH–SAP or SSP–SAP administration. DBH–SAP was previously validated as specifically ablating DBH^+^ neurons with no effect on neighbouring neurons^18,32,33^. Because a period of 2 weeks is necessary to eliminate DBH^+^ neurons using DBH–SAP^32^, mice were injected with SAP 1 week before the first sensitization and 2 weeks before the second challenge.

### Chemogenetic manipulation

Either CNO (Sigma, no. C0832, 1 mg kg^−1^, dissolved in 0.9% NaCl) or vehicle (0.9% NaCl) was injected intraperitoneally following expression of hM3D(Gq), hM4D(Gi) or mCherry in bilateral nTS or NA. The CNO concentration of 1 mg kg^−1^ used was effective^34–36^ and without apparent non-specific effects^37,38^. For hM4D(Gi) inhibition, CNO was injected 1 h before every of the second to fourth challenge; for hM3D(Gq) activation, CNO was injected in place of the fourth HDM challenge. Mice were euthanized 24 h later for flexiVent assay.

### Airway hyperreactivity assayed by flexiVent

Anaesthetized mice were paralysed with acepromazine (10 mg kg^−1^, intraperitoneal injection). Mice were tracheotomized with a 20 G sterile catheter and attached to a flexiVent pulmonary mechanics apparatus (SCIREQ). Mice were ventilated at 9 ml kg^−1^ tidal volume and a frequency of 150 beats min^−1^. The weight of each animal was entered into flexiVent at the start of each round of assay. Pre-scans were carried out as part of the flexiVent programme, allowing for the calculation of lung size. Positive end-expiratory pressure was set at 300 mm H_2_O. The nebulizer was activated for 10 s to deliver each dose of methacholine (0, 6, 12 or 24 mg ml^−1^, dissolved in 0.9% NaCl). The Rrs and Ers of the respiratory system were determined in response to aerosolized methacholine challenges, and the mean maximal elastance and resistance of 12 measurements by dose were then calculated. Statistical analysis at each methacholine concentration was performed separately.

### Tissue processing and flow cytometry

Anaesthetized mice were injected with AF700-counjugated CD45 (BioLegend, no. 103128, 10 µg per mouse) by intravenous injection to distinguish circulating immune cells and resident immune cells within the lung. Mice were euthanized 5 min later for lung harvest. Whole lungs were mechanically dissociated in GentleMACS C tubes (Miltenyi Biotec) containing 5 ml of RPMI 1640 (Thermo Scientific) with 10% fetal bovine serum, 1 mM HEPES (Life Technology), 1 mM MgCl_2_ (Life Technology), 1 mM CaCl_2_ (Sigma), 0.525 mg ml^−1^ collagenase/dispase (Roche) and 0.25 mg DNase I (Roche) by running the mouse lung 1-2 program on GentleMACS (Miltenyi Biotec). Lung pieces were then digested by shaking at around 150 rpm for 30 min at 37 °C. Following incubation, lung pieces were mechanically dissociated further using the mouse lung 2-1 program on GentleMACS, followed by straining through a 70 μm filter. Red blood cells were removed by the addition of 1 ml of RBC lysis buffer (BioLegend) to each tube and incubation at room temperature for 1 min. Single-cell suspensions were pelleted (1,500 rpm, 4 °C, 5 min), counted with a haemocytometer and diluted to around 1 × 10^6^ cells ml^−1^. Diluted cells were stained with Fc blocking antibody (5 mg ml^−1^, BD) before incubation with a surface marker antibody cocktail. For lung myeloid tissue, the following antibodies were used: 1:100 BV605-conjugated anti-F4/80 (BioLegend, no. 123133), 1:500 BV510-conjugated anti-CD45 (BioLegend, no. 110741), 1:1,000 APC-conjugated anti-CD11c (BioLegend, no. 117310), 1:1,000 PE-Cy7-conjugated anti-Ly6G (BioLegend, no. 560601) and 1:2,000 PE-CF594-conjugated anti-CD11b (BioLegend, no. 101256). For lung lymphoid tissue, the following antibodies were used: 1:200 FITC-conjugated anti-CD45 (BioLegend, no. 103108), 1:100 APC-Cy7-conjugated anti-IL-7Ra (BioLegend, no. 135040), V450-conjugated Lineage mix (1:200 anti-CD19, TONBO, no. 50-201-4944), 1:500 anti-CD11c (TONBO, no. 50-201-4937), 1:500 anti-F4/80 (TONBO, no. 50-201-4978), 1:100 anti-NK1.1 (BD, no. 560524), 1:100 anti-TER119 (BD, no. 560504), 1:100 anti-TCR gamma delta (Invitrogen, no. 48-5711-82), 1:100 BV510-conjugated anti-ST2 (BD, no. 745080), 1:200 PE-Cy7-conjugated anti-TCR-beta (BioLegend, no. 109222), 1:100 BV604-conjugated anti-CD4 (BioLegend, no. 100548) and 1:2,000 PerCP-Cy5.5-conjugated anti-CD90.2 (BioLegend, no. 105338). Cells were then stained using live/dead dye (1:1,000, Ghost Dye Red 780 (TONBO, no. 13-0865-T100 for myeloid tissue, 1:500 and Ghost Dye Violet 450, TONBO, no. 13-0863-T100)) before fixing using BD Stabilizing Fixative and transfer to fluorescent activated cell sorting tubes. Flow cytometry was analysed on a BD FACS Canto RUO - ORANGE analyser with three lasers (405, 488 and 640 nm) at the Flow Cytometry Core at VA San Diego Health Care System and San Diego Veterans Medical Research Foundation. All data were further analysed and plotted with FlowJo software (Tree Star). Eosinophils, group 2 innate lymphoid cells and T-helper 2 cells were gated on live, resident CD45^+^ singlets.

### Isolation of nTS and NA nuclei

Mice were euthanized using CO_2_ inhalation. For nTS, brainstems were acutely harvested from either (1) 4 groups of adult wild-type naive mice (*n* = 4 biological repeats, two males in each group); (2) mice at 1.5 h following the fourth saline treatment (2 males in the group); or (3) mice at 1.5 h following the fourth HDM challenge (2 males in the group). The nTS was visualized by microscopy and harvested based on anatomical landmarks. The reason for using males was based on our observation that males show less *Fos* background in saline control groups compared with females, providing a more consistent baseline for our study. Similarly, male mice were used in multiple, recently published single-cell RNA-seq datasets^14,15,27,39^. For NA, brainstems were acutely harvested from adult *Chat*-*cre;* *CAG-Sun1/sfGFP* mice (*n* = 7, 4 males and 3 females). NA was identified by nucleus-localized GFP fluorescence signals based on anatomical landmarks while avoiding DMV and 12N regions that also express *Chat*. NA samples from 7 mice were pooled for the snRNA-seq experiment. One pooled sample was assayed similarly in the other two studies with single-cell/-nucleus RNA-seq of the NA^12,13^. Dissected tissues were placed in liquid N_2_ immediately and either stored at −80 °C or sent directly for nuclei isolation.

On the day of nuclei dissociation, dissected tissues were transferred into 1 ml of douncing buffer (0.25 M sucrose, 25 mM KCl, 5 mM MgCl_2_, 10 mM Tris-HCl pH 7.5, 1 mM DTT (no. D9779, Sigma) and 1× cOmplete EDTA-free protease inhibitor (no. 05056489001, Roche, DB-DP, 0.1% Triton-X)). Pestled samples were filtered with 30 μm CellTrics and transferred to prechilled low-bind Eppendorf tubes. Samples were spun and sequentially resuspended in douncing buffer, permeabilization buffer (5% IGEPAL-CA630, no. I8896, Sigma, 0.2% DTT, 1 mM cOmplete EDTA-free protease inhibitor and 1× PBS) and tagmentation buffer (66 mM Tris-acetate pH 7.8 (no. BP-152, Thermo Fisher Scientific)), 132 mM K-acetate (no. P5708, Sigma), 20 mM Mg-acetate (no. M2545, Sigma) and 32 mM DMF (no. DX1730, Millipore) and counted using a haemocytometer.

### snRNA-seq and data analysis

Single-nucleus RNA sequencing experiments were carried out by the Center for Epigenomics, UCSD. Nuclei were processed into complementary DNA libraries using the Chromium Single Cell 3’ v3 kit (10X Genomics) and sequencing was carried out on the NovaSeq (Illumina) platform. The CellRanger software package from 10X Genomics (v3.0.2) was used to align raw reads onto the mouse reference genome (GRCm38) and generate the feature-barcode matrix. CellBender (v0.3.0)^40^ was then used to remove technical artefacts and ambient RNA to produce improved estimates of gene expression. The R package Seurat (v4.0)^29^ was then used to perform data quality control, normalization, principal components analysis, UMAP generation and differential gene expression testing. Nuclei with above 5% mitochondrial reads and greater than 2,000 unique genes were considered high-quality cells and were filtered for further analyses, following the filtering criteria commonly used in neuronal snRNA-seq studies including those on brainstem neurons^12–14^. In addition, DoubletFinder (v2.0)^41^ was used to remove doublets and SCTransform was used to normalize feature expression. Harmony (v1.2.0)^42^ was used to integrate individual datasets across three conditions (naive, saline and HDM). In total 42,157 nuclei were recovered, including 39,626 neurons. We then extracted nTS data from baseline naive condition (*n* = 4 biological repeats, *n* = 2 mice in each group) to profile marker gene expression across all nTS clusters. To determine dimensions for optimized clustering, we tested up to 50 principal components and evaluated the optimal cutoff using an elbow plot. In our study, we settled on using the first 20 principal components for clustering and projection with both UMAP and *t*-distributed stochastic neighbour embedding. We also tested a range of clustering resolutions (from 0.1 to 2.0) that were evaluated with clustree (v0.5.1; https://github.com/lazappi/clustree)^43^. In this dataset the resolution was set to 1.0, resulting in 25 interim clusters. We plotted a density UMAP using geom_density_2d and stat_density_2d (https://ggplot2.tidyverse.org/reference/geom_density_2d.html) from ggplot2 (v3.3.2)^44^ for visual identification of high-density regions that represent potential unique cell populations. Using these two methods, coupled with manual inspection of top markers, we combined several clusters with shared markers to ensure that annotated clusters would show unique transcriptional profiles, resulting in 18 distinct clusters (further details in Supplementary Note 1, Supplementary Fig. 2a–i and Supplementary Tables 1 and 2). Following combination, we reordered cluster ID based on the number of cells in each cluster and renumbered the largest cluster as cluster 1. For rigorous definition of marker genes for each cluster, we screened each cluster’s top marker genes (Seurat, FindAllMarkers) using ViolinPlot, FeaturePlot and DotPlot. Using the Seurat default dotplot setting (R package Seurat (v4.0)^29^, percentage expressed was plotted based on the actual percentage of cells expressing selected marker genes in a given cluster. Average expression was plotted based on the average normalized single-cell expression value, using the Seurat default dotplot setting (R package Seurat v4.0)^29^, with maximum average expression threshold set at 2.5 and everything higher set to this; and with minimum average expression threshold set at −2.5 and everything lower set to this. The colour bar shown on the right of the plot represents the range of scaled normalized expression values for the genes shown in that plot. We provide a list of the top 100 marker genes of nTS clusters in Supplementary Table 3.

For NA, 10,072 nuclei were recovered, including 7,664 neurons. Using *Chat* as a positive control gene to identify NA neurons, we removed clusters not showing *Chat* expression from ViolinPlot, FeaturePlot and DotPlot. We then followed the same pipeline given above to process our data and used Harmony (v1.2.0)^42^ to integrate our data with two published NA single-cell datasets^12,13^. We provide a list of the top 100 marker genes in Supplementary Table 4.

### qPCR

Total RNA was extracted from lungs using Trizol (Invitrogen) and the RNeasy Mini RNA extraction kit (Qiagen). qPCR with reverse transcription was then performed to obtain corresponding cDNA using THE iScript Select cDNA Synthesis Kit (Bio-Rad). qPCR was performed with the CFX ConnectTM system (Bio-Rad) using SYBR Green (Bio-Rad). At least three technical and three biological replicates were performed for each gene. Primer sequence: 5′-CGGCCAGGTCATCACTATTGGCAAC-3′, 5′-GCCACAGGATTCCATACCCAAGAAG-3′ for *Actb* (*β-actin*); 5′-TGACTCAATCTGCGTGCCTT-3′, 5′-AGGCCTTCTTTTGGCAGGTT-3′ for *Muc5ac*; 5′-GGTCTCAACCCCCAGCTAGT-3′, 5′-GCCGATGATCTCTCTCAAGTGAT-3′ for *Il4*; 5′-CCTCTTCGTTGCATCAGGGT-3′, 5′-GATCCTCCTGCGTCCATCTG-3′ for *Il5*; 5′-AAAGCAACTGTTTCGCCACG-3′, 5′-CCTCTCCCCAGCAAAGTCTG-3′ for *Il13*.

### Plethysmography

Ventilatory parameters during normoxia was measured in unrestrained male mice using a whole-body barometric plethysmograph modified for continuous flow^45–47^. Flow was maintained constant through the chamber while a pressure transducer (45 mMP with 2 cm H_2_O diaphragm, Validyne) recorded changes attributable to the warming and expansion of inhaled gases. Mice were weighed and sealed into an individual plethysmograph chamber along with a temperature and humidity probe (Thermalert TH5, Physitemp). A constant gas flow (335 ml min^−1^) was delivered using a rotameter (no. 603, Matheson) and measured with a flow meter (Sables System International, Inc.) upstream of the chamber. Gases exited the chamber through a valve and into a vacuum pump (Model 25, Precision Scientific Co.) to isolate pressure changes from respiration in the chamber during constant flow with high input and output impedances. All ventilatory parameters were recorded on an analogue-digital acquisition system (PowerLab 8SP, AD Instruments) and analysed with LabChart 8-Pro Software, sampling at a rate of 1 kHz. Mice were allowed to acclimatize to the chamber and constant air flow for 45 min (normoxia, 21% of O_2_) and were then exposed to a 5 min challenge of hypoxic gas (10% O_2_) to test responsiveness; they were then exposed to normoxic gas for 15 min. A minimum of 30 s between 10 and 15 min of normoxia exposure was analysed. Respiratory frequency (breaths min^−1^) was measured from cyclic peaks in the plethysmograph pressure pulses, and tidal volume (ml) was measured from calibration pulses using equations from ref. ^48^. Minute ventilation (${\dot{{\rm{V}}}}_{{\rm{i}}}$, ml min^−1^ kg) was calculated from respiratory frequency and tidal volume and normalized to body mass ventilation. Oxygen consumption $({\dot{{\rm{V}}}}_{{{\rm{O}}}_{2}})$ and carbon dioxide production $({\dot{{\rm{V}}}}_{{{\rm{CO}}}_{2}})$ were calculated by recording inspired and expired oxygen and carbon dioxide fractions using an O_2_/CO_2_ analyser. The ratio of ${\dot{{\rm{V}}}}_{{\rm{i}}}\,/{\dot{{\rm{V}}}}_{{{\rm{O}}}_{2}}$ to ${\dot{{\rm{V}}}}_{{\rm{i}}}\,/{\dot{{\rm{V}}}}_{{{\rm{CO}}}_{2}}$ thus provides a more precise estimation of mouse ventilation without the confounding factors produced by changes in metabolic rate.

### CUBIC tissue clearing and LSFM

Clear, unobstructed brain/body imaging cocktails and computational analysis protocol (CUBIC) buffers were prepared accordingly^49^. Following sufficient tissue clearing in R1 buffer, tissues were embedded in 2% low-melting agarose and then incubated in R2 solution before imaging. Cleared samples were imaged using a Zeiss Z.1 light sheet fluorescence microscope (LSFM). Vagal ganglia were imaged using a ×5 objective (LSFM ×5/0.1 numerical aperture (NA)) and a 1.45 ×5 CLARITY specific chamber. Lung samples were imaged using a ×2.5 objective (LSFM ×2.5/0.1 NA) and a 1.45 ×2.5 CLARITY specific chamber (Translucence Biosystems).

### CTB488 injection

Fully anaesthetized mice were placed ventral side up on a stereotaxic frame. The extrathoracic trachea was exposed via a neck incision. The trachea was carefully lifted without bleeding. Using a 10 μl Hamilton glass micropipette fitted with a 32 G needle, 10 μl of CTB488 (1 mg ml^−1^, Thermo Scientific, no. C22841) was injected into the dorsal aspect of the trachea from both sides, about three to five cartilage rings caudal to the larynx. Sutured mice were allowed to recover on a thermal pad before being returned to housing. Mice were euthanized 1 week following CTB488 injection for brainstem collection.

### Cannula implantation at bilateral NA

Fully anaesthetized mice were mounted on a stereotaxic frame. A midline incision was made to show bregma, the skull was cleaned with hydrogen peroxide and small holes were drilled through the skull at the designated stereotaxic coordinates of NA (6.7 mm caudal to bregma, 1.25 mm lateral to midline, 4.25 mm under the dura)^31^. A bilateral guide cannula affixed with two units of 26 G stainless-steel tubing (P1 Technologies, Inc.) was stereotaxically implanted 0.5 mm above the NA region. Guide cannulae were secured to the skull using superglue and dental cement. A matching dummy cannula were inserted into the guide cannula and secured with a dust cap to ensure guide cannula patency. Mice were allowed to recover in their home cages for 1 week before challenge and drug delivery. On the day of flexiVent assay, starting from baseline measurement, 1 μl of prazosin (0.4 mg ml^−1^ in ultrapure water, no. P7791, Sigma), terazosin (0.4 mg ml^−1^ in ultrapure water, no. T4680, Sigma) or ultrapure water control was consecutively microinjected into bilateral NA through the guided cannula using a two-channel syringe pump (no. R462, RWD).

### AAV infection of vagal ganglia

The vagal ganglia of anesthetized mice were surgically exposed by making an incision along the neck. A micropipette containing 200 nl of AAV2/9-syn-flex-GFP (2 × 10^13 ^gc ml^−1^; Boston Children’s Hospital Vector Core) was inserted into bilateral vagal ganglia. Mice were euthanized 3 weeks later for harvesting of vagal ganglia and brainstem.

### Statistics and reproducibility

Statistical analyses were calculated with Microsoft Excel and performed using Prism (GraphPad), with statistical tests and sample sizes reported in figure legends. Data in graphs are presented as mean ± s.e.m. and statistical tests are two-sided, unless otherwise indicated. All replicates were biological, unless otherwise indicated. All representative images are from at least three independent experiments, and details are described in figure legends. Sample sizes were determined based on previous expertise and publications in the field. Exact sample sizes are described in each figure legend. Investigators were blinded to group allocations for FOS antibody staining and flexiVent experiments associated with Figs. 1 and 3–6 and Extended Data Figs. 1–3 and 6–10; group allocation was not blinded in other experiments. Significance is defined as *P* < 0.05, with significance annotations of *P < 0.05, **P < 0.01, ***P < 0.001 and ****P < 0.0001. Absence of significant differences (*P* > 0.05) is indicated by NS (not significant).

### Materials availability

All reagents and materials used in this study are commercially available.

### Reporting summary

Further information on research design is available in the Nature Portfolio Reporting Summary linked to this article.

## Online content

Any methods, additional references, Nature Portfolio reporting summaries, source data, extended data, supplementary information, acknowledgements, peer review information; details of author contributions and competing interests; and statements of data and code availability are available at 10.1038/s41586-024-07608-5.

### Supplementary information

Supplementary Information This file contains Supplementary Note 1 and legends for Supplementary Figs. 1 and 2 and Tables 1–4.

Reporting Summary

Supplementary Fig. 1 Gating strategies used for cell sorting. (**a**) Gating strategies to determine the percentage of eosinophils (Eos) from whole lungs presented on Extended Data Figs. 1c, 3t, 6y and 8r. Red colored boxes indicate cells used for following analysis. AM, alveolar macrophage. (**b**) Gating strategies to determine the percentage of innate lymphoid cells (ILC2s) and T-helper type 2 cells (Th2) from whole lungs presented on Extended Data Figs. 1b,1d,3s,3u,6x,6z,8q and8s. Colored boxes indicate cells used for following analysis.

Supplementary Fig. 2 Clustree output and Density plot of the integrated nTS dataset. (**a**) Output from clustree for the different clustering resolutions using integrated nTS dataset. In this dataset, the resolution was set to 1.0 (indicated by arrowhead). (**b**-**h**) UMAP plots of the integrated nTS dataset from Resolution 0.4 to Resolution 1.0 (see Supplementary Note 1 for details). (**i**) Density UMAP plot of the integrated dataset.

Supplementary Table 1 Table of Top 200 differentially expressed marker genes of nTS Cluster 18 at Resolution 0.9 and Cluster 10 at Resolution 1.0. Marker genes for both clusters from “FindAllMarkers” analysis were sorted by adjusted p-value, following the default Seurat pipeline and based on Bonferroni correction using all features in the dataset. Shared marker genes were highlighted in yellow. See Supplementary Note 1 for details.

Supplementary Table 2 Table of Top 200 differentially expressed marker genes of 25 interim nTS clusters at Resolution 1.0. Marker genes for each cluster from “FindAllMarkers” analysis are sorted by adjusted p-value, following the default Seurat pipeline and based on Bonferroni correction using all features in the dataset. Shared marker genes between Clusters 8 and 3; Clusters 18, 19 and 1; Clusters 20 and 17; Clusters 22 and 7; Clusters 23 and 10; Clusters 24 and 16 were highlighted in yellow. See Supplementary Note 1 for details.

Supplementary Table 3 Table of Top 100 differentially expressed marker genes of nTS clusters. Top 100 marker genes for each cluster from “FindAllMarkers” analysis as sorted by adjusted p-value, following the default Seurat pipeline and based on Bonferroni correction using all features in the dataset. Marker genes that were plotted in Fig. 2f were highlighted in yellow. Marker genes that were validated in Fig. 2g-t and Extended Data Fig. 4h-c’, as well as used for quantification in Fig. 3c were bolded in red.

Supplementary Table 4 Table of Top 100 differentially expressed genes of NA clusters. Top 100 marker genes for each cluster from “FindAllMarkers” analysis as sorted by adjusted p-value, following the default Seurat pipeline and based on Bonferroni correction using all features in the dataset. Marker genes that were plotted in Fig. 6b were highlighted in yellow.

### Source data

Source Data Fig. 1

Source Data Fig. 3

Source Data Fig. 4

Source Data Fig. 5

Source Data Fig. 6

Source Data Extended Data Fig. 1

Source Data Extended Data Fig. 2

Source Data Extended Data Fig. 3

Source Data Extended Data Fig. 4

Source Data Extended Data Fig. 5

Source Data Extended Data Fig. 6

Source Data Extended Data Fig. 7

Source Data Extended Data Fig. 8

Source Data Extended Data Fig. 9

Source Data Extended Data Fig. 10

## Data Availability

The publicly available mouse genome reference mm10 (GENCODE vM23/Ensembl 98) from 10X Genomics was used for snRNA-seq analysis. Raw and fully processed snRNA-seq data reported in this study have been deposited in the Gene Expression Omnibus and are publicly available under accession numbers GSE200003 (for nTS) and GSE211538 (for NA). Additional data related to this paper may be requested from the authors; requests should be made directly to xinsun@health.ucsd.edu. Source data are provided with this paper.

## References

[1] McAlexander, M. A., Gavett, S. H., Kollarik, M. & Undem, B. J. Vagotomy reverses established allergen-induced airway hyperreactivity to methacholine in the mouse. *Respir. Physiol. Neurobiol.* **212–214**, 20–24 (2015). 25842220 10.1016/j.resp.2015.03.007 PMC4827162

[2] Tränkner, D., Hahne, N., Sugino, K., Hoon, M. A. & Zuker, C. Population of sensory neurons essential for asthmatic hyperreactivity of inflamed airways. *Proc. Natl Acad. Sci. USA* **111**, 11515–11520 (2014). 25049382 10.1073/pnas.1411032111 PMC4128113

[3] Talbot, S. et al. Silencing nociceptor neurons reduces allergic airway inflammation. *Neuron* **87**, 341–354 (2015). 26119026 10.1016/j.neuron.2015.06.007 PMC4506220

[4] Zhao, Q. et al. A multidimensional coding architecture of the vagal interoceptive system. *Nature* **603**, 878–884 (2022). 35296859 10.1038/s41586-022-04515-5 PMC8967724

[5] McGovern, A. E. et al. Evidence for multiple sensory circuits in the brain arising from the respiratory system: an anterograde viral tract tracing study in rodents. *Brain Struct. Funct.* **220**, 3683–3699 (2015). 25158901 10.1007/s00429-014-0883-9

[6] Su, Y. et al. Identification of lung innervating sensory neurons and their target specificity. *Am. J. Physiol. Lung Cell. Mol. Physiol.* **322**, L50–L63 (2021). 34755535 10.1152/ajplung.00376.2021 PMC8721910

[7] Han, W. et al. A neural circuit for gut-induced reward. *Cell* **175**, 887–888 (2018). 30340046 10.1016/j.cell.2018.10.018

[8] Ran, C., Boettcher, J. C., Kaye, J. A., Gallori, C. E. & Liberles, S. D. A brainstem map for visceral sensations. *Nature* **609**, 320–326 (2022). 36045291 10.1038/s41586-022-05139-5 PMC9452305

[9] Hitomi, K. et al. Allergin-1 on mast cells suppresses house dust mite-induced airway hyperresponsiveness in mice. *Int. Immunol.* **30**, 429–434 (2018). 30169732 10.1093/intimm/dxy025

[10] Corry, D. B. et al. Interleukin 4, but not interleukin 5 or eosinophils, is required in a murine model of acute airway hyperreactivity. *J. Exp. Med.* **183**, 109–117 (1996). 8551213 10.1084/jem.183.1.109 PMC2192426

[11] Tomer, R., Ye, L., Hsueh, B. & Deisseroth, K. Advanced CLARITY for rapid and high-resolution imaging of intact tissues. *Nat. Protoc.* **9**, 1682–1697 (2014). 24945384 10.1038/nprot.2014.123 PMC4096681

[12] Veerakumar, A., Yung, A. R., Liu, Y. & Krasnow, M. A. Molecularly defined circuits for cardiovascular and cardiopulmonary control. *Nature* **606**, 739–746 (2022). 35650438 10.1038/s41586-022-04760-8 PMC9297035

[13] Coverdell, T. C., Abraham-Fan, R. J., Wu, C., Abbott, S. B. G. & Campbell, J. N. Genetic encoding of an esophageal motor circuit. *Cell Rep.* **39**, 110962 (2022). 35705034 10.1016/j.celrep.2022.110962 PMC9255432

[14] Ilanges, A. et al. Brainstem ADCYAP1^+^ neurons control multiple aspects of sickness behaviour. *Nature* **609**, 761–771 (2022). 36071158 10.1038/s41586-022-05161-7 PMC9492535

[15] Zhang, C. et al. Area postrema cell types that mediate nausea-associated behaviors. *Neuron* **109**, 461–472 (2021). 33278342 10.1016/j.neuron.2020.11.010 PMC7864887

[16] Tao, J. et al. Highly selective brain-to-gut communication via genetically defined vagus neurons. *Neuron* **109**, 2106–2115 (2021). 34077742 10.1016/j.neuron.2021.05.004 PMC8273126

[17] Lein, E. S. et al. Genome-wide atlas of gene expression in the adult mouse brain. *Nature* **445**, 168–176 (2007). 17151600 10.1038/nature05453

[18] Ritter, S., Bugarith, K. & Dinh, T. T. Immunotoxic destruction of distinct catecholamine subgroups produces selective impairment of glucoregulatory responses and neuronal activation. *J. Comp. Neurol.* **432**, 197–216 (2001). 11241386 10.1002/cne.1097

[19] Baptista, V., Zheng, Z. L., Coleman, F. H., Rogers, R. C. & Travagli, R. A. Characterization of neurons of the nucleus tractus solitarius pars centralis. *Brain Res.* **1052**, 139–146 (2005). 16005442 10.1016/j.brainres.2005.05.073 PMC3070946

[20] Le Brun, I. et al. Differential expression of Nk1 and NK3 neurokinin receptors in neurons of the nucleus tractus solitarius and the dorsal vagal motor nucleus of the rat and mouse. *Neuroscience* **152**, 56–64 (2008). 18222044 10.1016/j.neuroscience.2007.12.024

[21] Mailhot-Larouche, S. et al. Assessment of respiratory function in conscious mice by double-chamber plethysmography. *J. Vis. Exp.* **137**, 57778 (2018). 10.3791/57778 PMC6126452 30059019

[22] Roman, C. W., Derkach, V. A. & Palmiter, R. D. Genetically and functionally defined NTS to PBN brain circuits mediating anorexia. *Nat. Commun.* **7**, 11905 (2016). 27301688 10.1038/ncomms11905 PMC4912612

[23] Aklan, I. et al. NTS catecholamine neurons mediate hypoglycemic hunger via medial hypothalamic feeding pathways. *Cell Metab.* **31**, 313–326 (2020). 31839488 10.1016/j.cmet.2019.11.016 PMC9017597

[24] Travagli, R. A. & Anselmi, L. Vagal neurocircuitry and its influence on gastric motility. *Nat. Rev. Gastroenterol. Hepatol.* **13**, 389–401 (2016). 27251213 10.1038/nrgastro.2016.76 PMC5605144

[25] Chen, C. Y. et al. Extended allergen exposure in asthmatic monkeys induces neuroplasticity in nucleus tractus solitarius. *J. Allergy Clin. Immunol.* **108**, 557–562 (2001). 11590381 10.1067/mai.2001.118132

[26] Pincus, A. B., Adhikary, S., Lebold, K. M., Fryer, A. D. & Jacoby, D. B. Optogenetic control of airway cholinergic neurons. *Am. J. Respir. Cell Mol. Biol.* **62**, 423–429 (2020). 31899655 10.1165/rcmb.2019-0378MA PMC7110977

[27] Prescott, S. L., Umans, B. D., Williams, E. K., Brust, R. D. & Liberles, S. D. An airway protection program revealed by sweeping genetic control of vagal afferents. *Cell* **181**, 574–589 (2020). 32259485 10.1016/j.cell.2020.03.004 PMC7197391

[28] Sun, X. et al. A census of the lung: CellCards from LungMAP. *Dev. Cell* **57**, 112–145 (2022). 34936882 10.1016/j.devcel.2021.11.007 PMC9202574

[29] Hao, Y. et al. Integrated analysis of multimodal single-cell data. *Cell* **184**, 3573–3587 (2021). 34062119 10.1016/j.cell.2021.04.048 PMC8238499

[30] Bai, L. et al. Genetic identification of vagal sensory neurons that control feeding. *Cell* **179**, 1129–1143 (2019). 31730854 10.1016/j.cell.2019.10.031 PMC6916730

[31] Franklin, K. B. J. & Paxinos, G. *Paxinos and Franklin’s The Mouse Brain in Stereotaxic Coordinates* 4th edn, Vol. 1 (Academic, 2013).

[32] Wrenn, C. C., Picklo, M. J., Lappi, D. A., Robertson, D. & Wiley, R. G. Central noradrenergic lesioning using anti-DBH-saporin: anatomical findings. *Brain Res.* **740**, 175–184 (1996). 8973812 10.1016/S0006-8993(96)00855-4

[33] Ippoliti, R., Lendaro, E., Bellelli, A. & Brunori, M. A ribosomal protein is specifically recognized by saporin, a plant toxin which inhibits protein synthesis. *FEBS Lett.* **298**, 145–148 (1992). 1544437 10.1016/0014-5793(92)80042-F

[34] Chang, S. E., Todd, T. P., Bucci, D. J. & Smith, K. S. Chemogenetic manipulation of ventral pallidal neurons impairs acquisition of sign-tracking in rats. *Eur. J. Neurosci.* **42**, 3105–3116 (2015). 26469930 10.1111/ejn.13103 PMC4715659

[35] Robinson, S. et al. Chemogenetic silencing of neurons in retrosplenial cortex disrupts sensory preconditioning. *J. Neurosci.* **34**, 10982–10988 (2014). 25122898 10.1523/JNEUROSCI.1349-14.2014 PMC4131013

[36] Zhang, Z. et al. An excitatory circuit in the perioculomotor midbrain for non-REM sleep control. *Cell* **177**, 1293–1307 (2019). 31031008 10.1016/j.cell.2019.03.041

[37] Manvich, D. F. et al. The DREADD agonist clozapine N-oxide (CNO) is reverse-metabolized to clozapine and produces clozapine-like interoceptive stimulus effects in rats and mice. *Sci. Rep.* **8**, 3840 (2018). 29497149 10.1038/s41598-018-22116-z PMC5832819

[38] Smith, K. S., Bucci, D. J., Luikart, B. W. & Mahler, S. V. Dreadds: use and application in behavioral neuroscience. *Behav. Neurosci.* **135**, 89–107 (2021). 34060867 10.1037/bne0000433

[39] Ludwig, M. Q. et al. A genetic map of the mouse dorsal vagal complex and its role in obesity. *Nat. Metab.* **3**, 530–545 (2021). 33767443 10.1038/s42255-021-00363-1 PMC12009600

[40] Fleming, S. J. et al. Unsupervised removal of systematic background noise from droplet-based single-cell experiments using CellBender. *Nat. Methods* **20**, 1323–1335 (2023). 37550580 10.1038/s41592-023-01943-7

[41] McGinnis, C. S., Murrow, L. M. & Gartner, Z. J. DoubletFinder: doublet detection in single-cell RNA sequencing data using artificial nearest neighbors. *Cell Syst.* **8**, 329–337 (2019). 30954475 10.1016/j.cels.2019.03.003 PMC6853612

[42] Korsunsky, I. et al. Fast, sensitive and accurate integration of single-cell data with Harmony. *Nat. Methods* **16**, 1289–1296 (2019). 31740819 10.1038/s41592-019-0619-0 PMC6884693

[43] Zappia, L. & Oshlack, A. Clustering trees: a visualization for evaluating clusterings at multiple resolutions. *Gigascience* **7**, giy083 (2018). 30010766 10.1093/gigascience/giy083 PMC6057528

[44] Wickham, H. *Ggplot2: Elegant Graphics for Data Analysis* (Springer Science + Business Media, LLC, 2016).

[45] Moya, E. A. et al. Neuronal HIF-1α in the nucleus tractus solitarius contributes to ventilatory acclimatization to hypoxia. *J. Physiol.* **598**, 2021–2034 (2020). 32026480 10.1113/JP279331 PMC7230006

[46] Reid, S. G. & Powell, F. L. Effects of chronic hypoxia on MK-801-induced changes in the acute hypoxic ventilatory response. *J. Appl. Physiol. (1985)* **99**, 2108–2114 (2005). 16109826 10.1152/japplphysiol.01205.2004

[47] Jacky, J. P. A plethysmograph for long-term measurements of ventilation in unrestrained animals. *J. Appl. Physiol. Respir. Environ. Exerc. Physiol.* **45**, 644–647 (1978). 101497 10.1152/jappl.1978.45.4.644

[48] Drorbaugh, J. E. & Fenn, W. O. A barometric method for measuring ventilation in newborn infants. *Pediatrics* **16**, 81–87 (1955). 14394741 10.1542/peds.16.1.81

[49] Susaki, E. A. et al. Advanced CUBIC protocols for whole-brain and whole-body clearing and imaging. *Nat. Protoc.* **10**, 1709–1727 (2015). 26448360 10.1038/nprot.2015.085

