# Supplementary material for: Brainstem Dbh+ neurons control allergen-induced airway hyperreactivity
Source: Nature. 2024 Jul 10;631(8021):601–9. doi: 10.1038/s41586-024-07608-5 (PMC11254774; doi:10.1038/s41586-024-07608-5)
Supplement: Supplementary file 1 — This file contains Supplementary Note 1 and legends for Supplementary Figs. 1 and 2 and Tables 1–4. [file 41586_2024_7608_MOESM1_ESM.pdf]

nature portfolio

https://doi.org/10.1038/s41586-024-07608-5

Supplementary information

Brainstem *Dbh*$^{+}$ neurons control allergen-induced airway hyperreactivity

In the format provided by the authors and unedited

Brainstem *Dbh*$^{+}$ Neurons

Control Allergen-Induced Airway Hyperreactivity

Authors: Yujuan Su[1], Jinhao Xu[1,2], Ziai Zhu[1], Jisun Chin[1], Le Xu[1], Haoze Yu[1], Victoria Nudell[3], Barsha Dash[4], Esteban A. Moya[5], Li Ye[3], Axel Nimmerjahn[6], Xin Sun[1,2,*]

Affiliations:

[1]Department of Pediatrics, School of Medicine, University of California San Diego, La Jolla, CA 92093, USA

[2]Department of Biological Sciences, University of California San Diego, La Jolla, CA 92093, USA

[3]Department of Neuroscience, Scripps Research Institute, La Jolla, CA 92037, USA

[4]La Jolla Institute for Immunology, La Jolla, CA 92037, USA

[5]Division of Pulmonary, Critical Care & Sleep Medicine, Department of Medicine, University of California, San Diego, CA 92093, USA

[6]Waitt Advanced Biophotonics Center, The Salk Institute for Biological Studies, La Jolla, CA 92037, USA

*Correspondence author: xinsun@health.ucsd.edu

Supplementary Note 1. Details about how we reached the 18 distinct nTS clusters utilizing combined approaches of clustree, density plot, and manual plotting of all markers to ensure specificity.

Briefly, following clustree analysis (Supplementary Fig. 2a), we manually checked Resolutions 0.4-0.9 by plotting top marker genes, and found that they were under-clustered. As shown in Supplementary Fig. 2b-d, we ruled out Resolutions 0.4, 0.5 and 0.6 as the final resolution because Cluster 18 (Fig. 2b) in the final object has unique marker genes, as depicted in the dotplot in Fig. 2f. However, at these resolutions, Cluster 18 could not be separated out as a distinct cluster, it was inappropriately grouped with Cluster 9 at Resolution 0.4 (Supplementary Fig. 2b), with Cluster 10 at Resolution 0.5 (Supplementary Fig. 2c), or with Cluster 9 at Resolution 0.6 (Supplementary Fig. 2d). We also dismissed Resolutions 0.7 and 0.8 as Clusters 12 and 17 in the final object (Fig. 2b) should be separated clusters, each with distinct marker genes (Fig. 2f). However, at Resolutions 0.7 and 0.8, they were inappropriately combined into one cluster. Clusters 12 and 17 in the final object were inappropriately combined with Cluster 4 at Resolution 0.7 (Supplementary Fig. 2e), and with Cluster 5 at Resolution 0.8 (Supplementary Fig. 2f). We noticed the area of Cluster 10 at Resolution 1.0 (Supplementary Fig. 2h) is bigger than the area of Cluster 18 at Resolution 0.9 (Supplementary Fig. 2g). When we compared Top 200 marker genes of Cluster 18 at Resolution 0.9 and Cluster 10 at Resolution 1.0 as sorted by adjusted P value, we found 75% of their marker genes were shared between the two clusters (Supplementary Table 1, shared marker genes were highlighted in yellow), suggesting these extra cells did not have distinct enough gene signatures and should be combined to its nearby cluster, resulting in Cluster 10 at Resolution 1.0 (Supplementary Fig. 2h).

At Resolution 1.0, we ensured the uniqueness of 25 clusters by plotting top marker genes for each cluster. We identified clusters (Clusters 8 and 3; Clusters 18, 19 and 1; Clusters 20 and 17; Clusters 22 and 7; Clusters 23 and 10; Clusters 24 and 16) with shared marker genes (Supplementary Table 2, shared marker genes were highlighted in yellow) and manually combined these clusters. We then plotted a density UMAP using geom_density_2d and stat_density_2d (https://ggplot2.tidyverse.org/reference/geom_density_2d.html) from ggplot2 (v3.3.2) and confirmed visually distinguishable high-density regions suggestive of unique cell populations (Supplementary Fig. 2i). Supplementary Table 2 provided all the marker genes for each of the original 25 clusters from "FindAllMarkers" analysis and highlighted the shared marker genes between Clusters 8 and 3; Clusters 18, 19 and 1; Clusters 20 and 17; Clusters 22 and 7; Clusters 23 and 10; Clusters 24 and 16, facilitating reproducibility of our analysis.
