# Supplementary material for: Brainstem Dbh+ neurons control allergen-induced airway hyperreactivity
Source: Nature. 2024 Jul 10;631(8021):601–9. doi: 10.1038/s41586-024-07608-5 (PMC11254774; doi:10.1038/s41586-024-07608-5)
Supplement: Supplementary file 2 — Reporting Summary [file 41586_2024_7608_MOESM2_ESM.pdf]

nature portfolio

Corresponding author(s): Xin Sun

Last updated by author(s): Apr 20, 2024

Reporting Summary

Nature Portfolio wishes to improve the reproducibility of the work that we publish. This form provides structure for consistency and transparency in reporting. For further information on Nature Portfolio policies, see our Editorial Policies and the Editorial Policy Checklist.

Statistics

For all statistical analyses, confirm that the following items are present in the figure legend, table legend, main text, or Methods section.

n/a	Confirmed	
☐	☒	The exact sample size (*n*) for each experimental group/condition, given as a discrete number and unit of measurement
☐	☒	A statement on whether measurements were taken from distinct samples or whether the same sample was measured repeatedly
☐	☒	The statistical test(s) used AND whether they are one- or two-sided<br>*Only common tests should be described solely by name; describe more complex techniques in the Methods section.*
☐	☒	A description of all covariates tested
☐	☒	A description of any assumptions or corrections, such as tests of normality and adjustment for multiple comparisons
☐	☒	A full description of the statistical parameters including central tendency (e.g. means) or other basic estimates (e.g. regression coefficient) AND variation (e.g. standard deviation) or associated estimates of uncertainty (e.g. confidence intervals)
☐	☒	For null hypothesis testing, the test statistic (e.g. *F*, *t*, *r*) with confidence intervals, effect sizes, degrees of freedom and *P* value noted<br>*Give P values as exact values whenever suitable.*
☒	☐	For Bayesian analysis, information on the choice of priors and Markov chain Monte Carlo settings
☒	☐	For hierarchical and complex designs, identification of the appropriate level for tests and full reporting of outcomes
☒	☐	Estimates of effect sizes (e.g. Cohen's *d*, Pearson's *r*), indicating how they were calculated

Our web collection on statistics for biologists contains articles on many of the points above.

Software and code

Policy information about availability of computer code

Data collection	CellRanger package (v3.0.2), CellBender (v0.3.0), R package Seurat (v4.0), DoubletFinder (v2.0), Harmony (v1.2.0), clustree (v.0.5.1) and ggplot2 (v3.3.2).
Data analysis	All statistics were calculated using Microsoft Excel and performed using GraphPad Prism (GraphPad Software Inc., CA, USA). The overlaps between Fos signals and each of the 18 nTS cluster marker genes in the nTS, and between Fos signals and tdTom signals for Dbh-cre;Ai14 mice were quantified using Qupath. All flow data were analyzed and plotted with FlowJo software (Tree Star). Single nucleus data of nTS and NA were analyzed using CellRanger package (v3.0.2), CellBender (v0.3.0), R package Seurat (v4.0), DoubletFinder (v2.0), Harmony (v1.2.0), clustree (v.0.5.1) and ggplot2 (v3.3.2).

For manuscripts utilizing custom algorithms or software that are central to the research but not yet described in published literature, software must be made available to editors and reviewers. We strongly encourage code deposition in a community repository (e.g. GitHub). See the Nature Portfolio guidelines for submitting code & software for further information.

Data

Policy information about availability of data

All manuscripts must include a data availability statement. This statement should provide the following information, where applicable:

- Accession codes, unique identifiers, or web links for publicly available datasets
- A description of any restrictions on data availability
- For clinical datasets or third party data, please ensure that the statement adheres to our policy

The public available mouse genome reference mm10 (GENCODE vM23/Ensembl 98) from 10X Genomics was used for single-nucleus RNA sequencing analysis. Raw and fully processed single-nucleus RNA sequencing data reported in this study have been deposited in the Gene Expression Omnibus and are publicly available under the accession numbers: GSE200003 (for nTS) and GSE211538 (for NA). Additional data related to this paper may be requested from the authors. Request should be directly to xinsun@health.ucsd.edu. Source data are provided with this paper.

Human research participants

Policy information about studies involving human research participants and Sex and Gender in Research.

Reporting on sex and gender	N/A
Population characteristics	N/A
Recruitment	N/A
Ethics oversight	N/A

Note that full information on the approval of the study protocol must also be provided in the manuscript.

Field-specific reporting

Please select the one below that is the best fit for your research. If you are not sure, read the appropriate sections before making your selection.

☒ Life sciences ☐ Behavioural & social sciences ☐ Ecological, evolutionary & environmental sciences

For a reference copy of the document with all sections, see nature.com/documents/nr-reporting-summary-flat.pdf

Life sciences study design

All studies must disclose on these points even when the disclosure is negative.

Sample size	Sample sizes were determined based on previous expertise and publications in the field (for example, PMID: 25842220, 25049382, 26119026, 34755535). Exact sample sizes are described in each figure legend or Methods.
Data exclusions	Preestablished criteria was used for inclusion/exclusion of data from studies. For studies based on stereotaxtic injection or cannula implant, mice were excluded from data if post-hoc analysis of their injection/implant sites deemed them as not having appropriate injections or targeting in the regions of interest.
Replication	All replicates were biological, unless otherwise indicated. All figures depicting representative images were independently replicated at least three times, and details are described in figure legends.
Randomization	Mice were randomly assigned to control and experimental groups.
Blinding	Investigators were blinded to group allocations for FOS antibody staining and flexiVent experiments associated with Figs. 1, 3-6 and Extended Data Figs. 1-3 and 6-10; group allocation was not blinded in other experiments. Blinding for experiments involving RNAScope valadation for single-nucleus dataset or antibody validation after virus injection were not possible as the gene names, virus information and genotype of the mice are necessarily apparent in deciding the RNAscope probes or antibodies for analysis.

Reporting for specific materials, systems and methods

We require information from authors about some types of materials, experimental systems and methods used in many studies. Here, indicate whether each material, system or method listed is relevant to your study. If you are not sure if a list item applies to your research, read the appropriate section before selecting a response.

Materials & experimental systems

n/a	Involved in the study
☐	☒ Antibodies
☒	☐ Eukaryotic cell lines
☒	☐ Palaeontology and archaeology
☐	☒ Animals and other organisms
☒	☐ Clinical data
☒	☐ Dual use research of concern

Methods

n/a	Involved in the study
☒	☐ ChIP-seq
☐	☒ Flow cytometry
☒	☐ MRI-based neuroimaging

Antibodies

Antibodies used

For immunofluorescence staining: Primary antibodies used include rabbit anti-c-FOS (SYSY, 226 008, 1:300), rabbit anti-DBH (Sigma, AB1585, 1:300), rabbit anti-Dsred (Takara, 632496, 1:300), rabbit anti-VAChT (SYSY, 139 103, 1:300), mouse anti-alpha Smooth Muscle Actin-FITC (Sigma, F3777, 1:300), rabbit anti-TRPV1 (Alomone labs, ACC-030, 1: 300) and chicken anti-GFP (Abcam, ab13970, 1:300). Secondary antibodies used include goat anti-rabbit FITC, goat anti-rabbit Cy3, goat anti-rabbit Cy5 (all from Jackson Immuno Research Labs, all 1:300).
For flow cytometry: AF700-counjugated CD45 (BioLegend, 103128, 10 µg/mouse. For lung myeloid, the following antibodies were used: 1:100 BV605-conjugated anti-F4/80 (BioLegend, 123133); 1:500 BV510-conjugated anti-CD45 (BioLegend, 110741); 1:1000 APC-conjugated anti-CD11c (BioLegend, 117310); 1:1000 PE-Cy7-conjugated anti-Ly6G (BioLegend, 560601); 1:2000 PE-CF594-conjugated anti-CD11b (BioLegend, 101256), 1:1000, Ghost Dye Red 780 (TONBO, 13-0865-T100). For lung lymphoid, the following antibodies were used: 1:200 FITC-conjugated anti-CD45 (BioLegend, 103108); 1:100 APC-Cy7-conjugateed anti-IL-7Ra (BioLegend, 135040); V450-conjugated Lineage mix (1:200 anti-CD19 (TONBO, 50-201-4944) ; 1:500 anti-CD11c (TONBO, 50-201-4937); 1:500 anti-F4/80 (TONBO, 50-201-4978); 1:100 anti-NK1.1(BD, 560524); 1:100 anti-TER119 (BD, 560504); 1:100 anti-TCR gamma delta (Invitrogen, 48-5711-82)); 1:100 BV510-conjugated anti-ST2 (BD, 745080); 1:200 PE-Cy7-conjugated anti-TCR-beta (BioLegend, 109222); 1:100 BV604-conjugated anti-CD4 (BioLegend, 100548); 1:2000 PerCP-Cy5.5-conjugated anti-CD90.2 (BioLegend, 105338), Ghost Dye Violet 450 (TONBO, 13-0863-T100).

Validation

Primary and secondary antibodies are commercially available and validated by the manufacturers, quality control practices and previous publications.

For rabbit anti-c-FOS (SYSY, 226 003), validated by manufacturer and previous publications: https://www.sysy.com/product/226008
Remarks: This antibody is a chimeric antibody based on the monoclonal rat antibody clone 108B5. The constant regions of the heavy and light chains have been replaced by rabbit specific sequences. Therefore, the antibody can be used with standard anti-rabbit secondary reagents.
Previous publications: PMID:28430937, PMID:29398217, PMID:29804835, PMID:29887312, PMID:30225361, PMID:30528578, PMID:31031588, PMID:31097621, PMID:31133559, PMID:31227310, PMID:31232695, PMID:31402172, PMID:31738230, PMID:31925973, PMID:31952856, PMID:32005806, PMID:32277045, PMID:32457072, PMID:32519950, PMID:32692847

For rabbit anti-DBH (Sigma, AB1585), validated by manufacturer and previous publications: https://www.sigmaaldrich.com/US/en/product/mm/ab1585
Specificity: Recognizes Dopamine beta hydroxylase. AB1585 reacts with a single band on Western blots of bovine adrenal homogenates and stains only cells known to contain DBH, such as sympathetic neurons, adrenal medullary cells and central adrenergic neurons. Staining is abolished by pre-incubation with enzyme.
Previous publications: PMID:17099901, PMID:29109240, PMID:30095409, PMID:31757673

For rabbit anti-Dsred (Takara, 632496), validated by manufacturer and previous publications: https://www.takarabio.com/products/antibodies-and-elisa/fluorescent-protein-antibodies/red-fluorescent-protein-antibodies
Remarks: The Living Colors DsRed Polyclonal Antibody has been raised against DsRed-Express, a variant of Discosoma sp. red fluorescent protein. This antibody recognizes DsRed-Express, DsRed-Express2, DsRed-Monomer, mCherry, DsRed2, E2-Crimson, tdTomato, mStrawberry, and mBanana, and both N- and C-terminal fusion proteins containing these fluorescent proteins in mammalian cell lysates.
Previous publications: PMID:20575070, PMID:20878781, PMID:21452218, PMID:21713771, PMID:21935944, PMID:23224947, PMID:23605441, PMID:23696496, PMID:23749685, PMID:23749724, PMID:24715505, PMID:25232112, PMID:25339743, PMID:25556545, PMID:25913861, PMID:25978516, PMID:26234537, PMID:26399201, PMID:26400711, PMID:26586220, PMID:26587737, PMID:26762251, PMID:26889940, PMID:27065364, PMID:27422730, PMID:27494275, PMID:27532901, PMID:27683912, PMID:27693369, PMID:27743477, PMID:27916275, PMID:27997037, PMID:28032634, PMID:28041852, PMID:28123013, PMID:28211790, PMID:28235898, PMID:28285821, PMID:28323938, PMID:28340519, PMID:28343864, PMID:28457596, PMID:28462393, PMID:28472655, PMID:28472660, PMID:28489528, PMID:28495975, PMID:28502772, PMID:28561736, PMID:28632130, PMID:28641115, PMID:28689640, PMID:28701929, PMID:28712654, PMID:28752052, PMID:28768176, PMID:28781050, PMID:28781169, PMID:28785727, PMID:28802047, PMID:28815501, PMID:28821643, PMID:28823729, PMID:28826487, PMID:28842919, PMID:28844842, PMID:28858617, PMID:28885142, PMID:28888696, PMID:28921616, PMID:28925357, PMID:28984573, PMID:29022877, PMID:29022879, PMID:29055051, PMID:29071300, PMID:29077837, PMID:29107546, PMID:29149607, PMID:29224725, PMID:29225025, PMID:29276142, PMID:29307485, PMID:29346753, PMID:29395056, PMID:29396116, PMID:29398621, PMID:29400650, PMID:29412136, PMID:29466446, PMID:29486195, PMID:29500189, PMID:29513217, PMID:29522093, PMID:29528286, PMID:29547121, PMID:29553369, PMID:29555855, PMID:29561262, PMID:29576390, PMID:29606582, PMID:29621490, PMID:29664120, PMID:29673482, PMID:29691331, PMID:29719245, PMID:29728449, PMID:29747217, PMID:29760181, PMID:29773754, PMID:29806907, PMID:29847801, PMID:29910074, PMID:29917235, PMID:29961574, PMID:30078709, PMID:30116771, PMID:30136927, PMID:30157424, PMID:30174119, PMID:30197236, PMID:30205037, PMID:30230471, PMID:30232002, PMID:30245010, PMID:30245154, PMID:30255935, PMID:30257208, PMID:30266741, PMID:30270186, PMID:30282024, PMID:30282729,

PMID:30290179, PMID:30304401, PMID:30318411, PMID:30318414, PMID:30325514, PMID:30340041, PMID:30343334, PMID:30355484, PMID:30355627, PMID:30377226, PMID:30415995, PMID:30415997, PMID:30467079, PMID:30541071, PMID:30555002, PMID:30612231, PMID:30620045, PMID:30622165, PMID:30661739, PMID:30661796, PMID:30726729, PMID:30744968, PMID:30773368, PMID:30794158, PMID:30819798, PMID:30824323, PMID:30824354, PMID:30833511, PMID:30849972, PMID:30853556, PMID:30855229, PMID:30865587, PMID:30880014, PMID:30886013, PMID:30893588, PMID:30893593, PMID:30907928, PMID:30917309, PMID:30958266, PMID:30970247, PMID:30994458, PMID:31006648, PMID:31006650, PMID:31056285, PMID:31074796, PMID:31099753, PMID:31116972, PMID:31155354, PMID:31209173, PMID:31225795, PMID:31242429, PMID:31248728, PMID:31291586, PMID:31300524, PMID:31320449, PMID:31390558, PMID:31390563, PMID:31390571, PMID:31392919, PMID:31395429, PMID:31398341, PMID:31427403, PMID:31442201, PMID:31461644, PMID:31533036, PMID:31539496, PMID:31577916, PMID:31618460, PMID:31645458, PMID:31664163, PMID:31685650, PMID:31687928, PMID:31693884, PMID:31736464, PMID:31746739, PMID:31747611, PMID:31759144, PMID:31786064, PMID:31793875, PMID:31810837, PMID:31866223, PMID:31891351, PMID:31939737, PMID:31955847, PMID:31955990, PMID:32012264, PMID:32125273, PMID:32170734, PMID:32286225, PMID:32314957, PMID:32314964, PMID:32367800, PMID:32381649, PMID:32383444, PMID:32452384, PMID:32463364, PMID:32568072, PMID:32573436, PMID:32641403, PMID:32675174

For rabbit anti-VAChT (SYSY, 139 103), validated by manufacturer and previous publications: https://sysy.com/product/139103
Specificity and Remarks: K.O. validated, PMID: 24027290. This antibody detects the glycosylated and unglycosylated protein and is an excellent marker for cholinergic axons.
Previous publications: PMID:19226511, PMID:26779909, PMID:27707979, PMID:29311772, PMID:29514065, PMID:29574885, PMID:30471164, PMID:30773633, PMID:31506825, PMID:31697941

For mouse anti-alpha Smooth Muscle Actin-FITC (Sigma, F3777), validated by manufacturer and previous publications: https://www.sigmaaldrich.com/US/en/product/sigma/f3777
Specificity: Monoclonal Anti-Actin, α-Smooth Muscle specifically recognizes the α-smooth muscle isoform of actin (42 kDa) by ELISA and immunoblotting. It does not react with the other major actin isoforms present in fibroblasts or epithelial cells (β and γ-cytoplasmic), striated muscle (α-sarcomeric), myocardium (α-myocardial), or γ-smooth muscle isoform.
Previous publications: PMID:23720424, PMID:27504807, PMID:27867035, PMID:29506142, PMID:30016619, PMID:30054204, PMID:30174297, PMID:30645973, PMID:30686582, PMID:30830222, PMID:31883835, PMID:31935371, PMID:31968248, PMID:32648540

For rabbit anti-TRPV1 (Alomone labs, ACC-030), validated by manufacturer and previous publications: https://www.alomone.com/p/anti-trpv1/ACC-030
Specificity: Knockout validation of Anti-TRPV1 (VR1) Antibody in mouse adipose tissue lysate. Western blot analysis of mouse adipose tissue lysate using Anti-TRPV1 (VR1) Antibody (#ACC-030). TRPV1 is not detected in TRPV1-/- animals. PMID: 25849380.
Previous publications: PMID: 37986234, PMID: 38226500, PMID: 26043006, PMID: 25787958

For chicken anti-GFP (Abcam, ab13970), validated by manufacturer and previous publications: https://www.abcam.com/products/primary-antibodies/gfp-antibody-ab13970.html
Specificity: Our GFP antibody does cross-react with the many fluorescent proteins that are derived from the jellyfish Aequorea victoria. These are all proteins that differ from the original GFP by just a few point mutations (EGFP, YFP, mVenus, CFP, BFP etc.).
Previous publications:
PMID: 36413988, PMID: 36527899, PMID: 36635457, PMID:35667851, PMID:34997978

Jackson Immuno Research Labs: Based on immunoelectrophoresis and/or ELISA, the antibody reacts with whole molecule of host Ig. It also reacts with the light chains of other host species immunoglobulins. No antibody was detected against non-immunoglobulin serum proteins. The antibody has been tested by ELISA and/or solid-phase adsorbed to ensure minimal cross-reaction with non-host species such as chicken, guinea pig, syrian hamster, goat, horse, human, mouse, rabbit and rat serum proteins, but it may cross-react with immunoglobulins from other species. Whole IgG antibodies are isolated as intact molecules from antisera by immunoaffinity chromatography. They have an Fc portion and two antigen binding Fab portions joined together by disulfide bonds and therefore they are divalent. The average molecular weight is reported to be about 160 kDa. The whole IgG form of antibodies is suitable for the majority of immunodetection procedures and is the most cost effective.

Animals and other research organisms

Policy information about studies involving animals; ARRIVE guidelines recommended for reporting animal research, and Sex and Gender in Research

Laboratory animals	Animals were maintained under constant environmental conditions (temperature in rooms is between 68-72 F and humidity is 30-70%) with food and water provided ad libitum in a 12-h light–dark cycle. Adult mice from strains: C57BL/6J (JAX 000664), c-Kitw-sh/w-sh (JAX 030764), Fos2A-iCreER (TRAP2, JAX 030323), Rosa-lxl-tdTomato (Ai14, JAX 007914), Rosa-lxl-DTR (JAX 016603), Th-cre (JAX 008601), Chat-cre (JAX 031661), CAG-Sun1/sfGFP (JAX 030952) and Rosa-Zsgreen (JAX 007906) were purchased from the Jackson lab. Dbh-cre (MMRRC 036778) was purchased from the Mutant Mouse Resource and Research Center (MMRRC). All the cre lines we used in this study were kept in B6 background. All cre lines were kept in B6 background and were viable and fertile with no detectable abnormal phenotypes. Both male and female mice were used for experiments. Mice were at least six-week-old when subjected to HDM challenge, stereotaxic injection or surgery.
Wild animals	This study did not involve wild animals.
Reporting on sex	For the nTS snRNA-seq, we harvested nTS from B6 males. The reason for using males for the nTS experiment is because we found that compared to females, males show less FOS background in saline control groups, and therefore offer a more consistent baseline. Aside from nTS snRNA-seq, FOS-staining and Fos2A-iCreER (TRAP2)-reporter tracing experiments, we performed all other functional studies using both males and females.

Field-collected samples	This study did not involve samples collected in the field.
Ethics oversight	All mice were housed, and all experimental procedures were carried out in American Association for Accreditation of Laboratory Animal Care-certified laboratory animal facilities at the University of California, San Diego. All animal procedures were approved by the Institutional Animal Care and Use Committee at the University of California, San Diego.

Note that full information on the approval of the study protocol must also be provided in the manuscript.

Flow Cytometry

Plots

Confirm that:

☒ The axis labels state the marker and fluorochrome used (e.g. CD4-FITC).

☒ The axis scales are clearly visible. Include numbers along axes only for bottom left plot of group (a 'group' is an analysis of identical markers).

☒ All plots are contour plots with outliers or pseudocolor plots.

☒ A numerical value for number of cells or percentage (with statistics) is provided.

Methodology

Sample preparation	Three days after last challenge, mice were anesthetized (100 mg/kg ketamine and 10 mg/kg xylazine, intraperitoneal injection) and then injected with AF700-conjugated CD45 through intravenous injection to distinguish circulating immune cells and immune cells resident within the lung. Mice were euthanized 5 minutes later for lung harvest. Whole lungs were mechanically dissociated in GentleMACS C tubes (Miltenyi Biotec) containing 5 ml of PRMI 1640 (Thermo Scientific) with 10 % FBS, 1 mM HEPES (Life Technology), 1 mM MgCl2 (Life Technology), 1 mM CaCl2 (Sigma-Aldrich), 0.525 mg/ml collagenase/dispase (Roche) and 0.25 mg DNase I (Roche) by running mouse lung 1-2 program on GentleMACS (Miltenyi Biotec). Lung pieces were then digested by shaking (~150 rpm) for 30 minutes at 37°C. After incubation, Lung pieces were mechanically dissociated further using mouse lung 2-1 program on GentleMACS, followed by straining through a 70 mm filter. Red blood cells were removed by adding 1 mL RBC lysis buffer (Biolegend) to each tube and incubate at room temperature for 1 minute. The single-cell suspensions from above were then pelleted (1500 rpm, 4°C, 5 minutes), counted with hemocytometer and diluted to around 1 × 106 cells per ml. They were stained with Fc blocking antibody (5 mg/ml, BD) at 4°C for 30 minutes. The cells were washed with DPBS and then incubated with surface marker antibody cocktail for 30 minutes at 4°C.
Instrument	Flow cytometry was analyzed on BD FACS Canto RUO - ORANGE with three lasers (405 nm, 488 nm and 640 nm) using the Flow Cytometry Core at the VA San Diego Health Care System and the San Diego Veterans Medical Research Foundation.
Software	All data were further analyzed and plotted with FlowJo software (Tree Star).
Cell population abundance	Live cells were determined by staining using live/dead dye (1:1000, Ghost Dye Red 780 for myeloid, 1:500, Ghost Dye Violet 450 for lymphoid).
Gating strategy	Eosinophils, innate lymphoid cells (ILC2s) and Th2 cells were gated on live, resident CD45+ singlets. Eosinophil population were gated as F4/80+, Ly6G-, CD11c-, CD11b+ from myeloid. ILC2s were gated as CD4-, TCRb-, ST2+, IL-7Ra+ population from lymphoid. Th2 cells were gated as CD4+, TCRb+, CD90.2+, ST2+ population from lymphoid.

☒ Tick this box to confirm that a figure exemplifying the gating strategy is provided in the Supplementary Information.
