# Supplementary figures and images for: Brainstem Dbh+ neurons control allergen-induced airway hyperreactivity
Source: Nature. 2024 Jul 10;631(8021):601–9. doi: 10.1038/s41586-024-07608-5 (PMC11254774; doi:10.1038/s41586-024-07608-5)

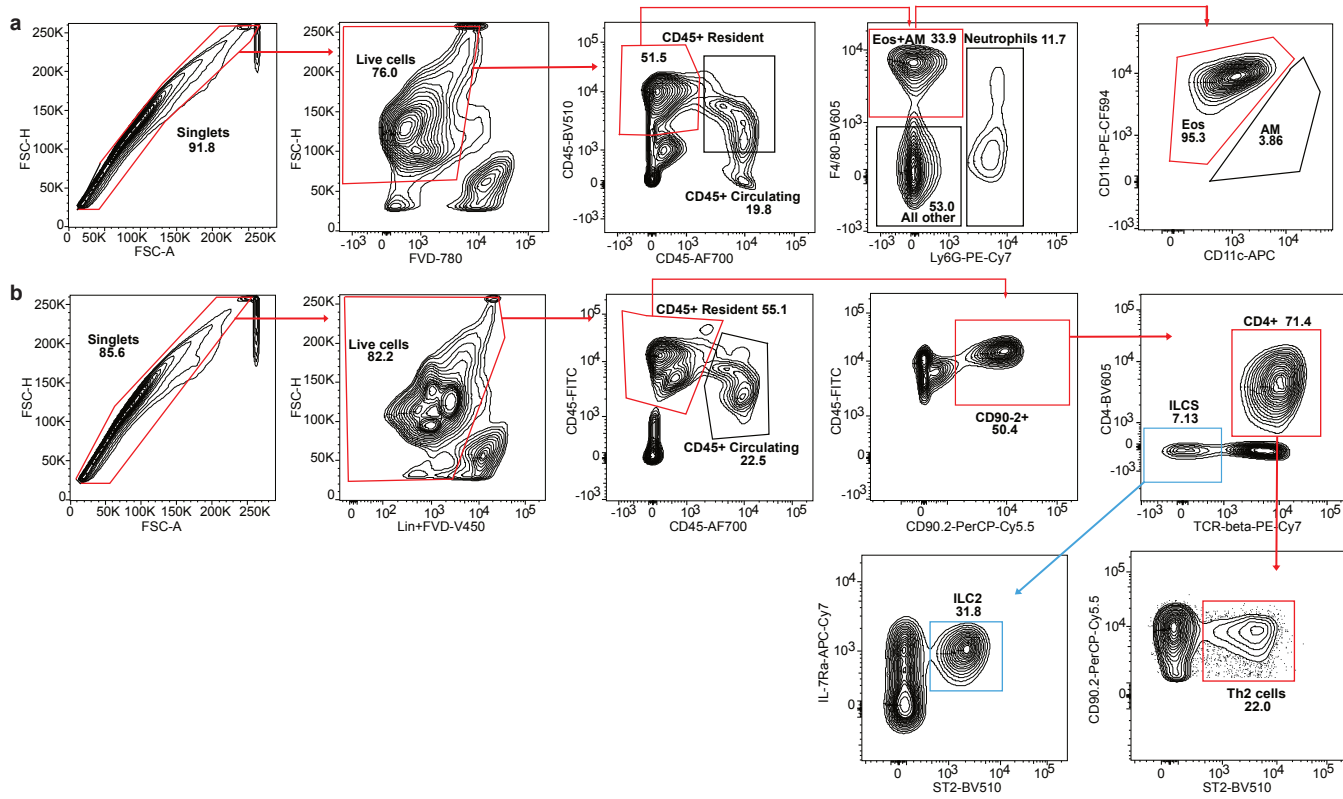

Supplement: Supplementary file 3 — Gating strategies used for cell sorting. (a) Gating strategies to determine the percentage of eosinophils (Eos) from whole lungs presented on Extended Data Figs. 1c, 3t, 6y and 8r. Red colored boxes indicate cells used for following analysis. AM, alveolar macrophage. (b) Gating strategies to determine the percentage of innate lymphoid cells (ILC2s) and T-helper type 2 cells (Th2) from whole lungs presented on Extended Data Figs. 1b,1d,3s,3u,6x,6z,8q and8s. Colored boxes indicate cells used for following analysis. [file 41586_2024_7608_MOESM3_ESM.pdf]

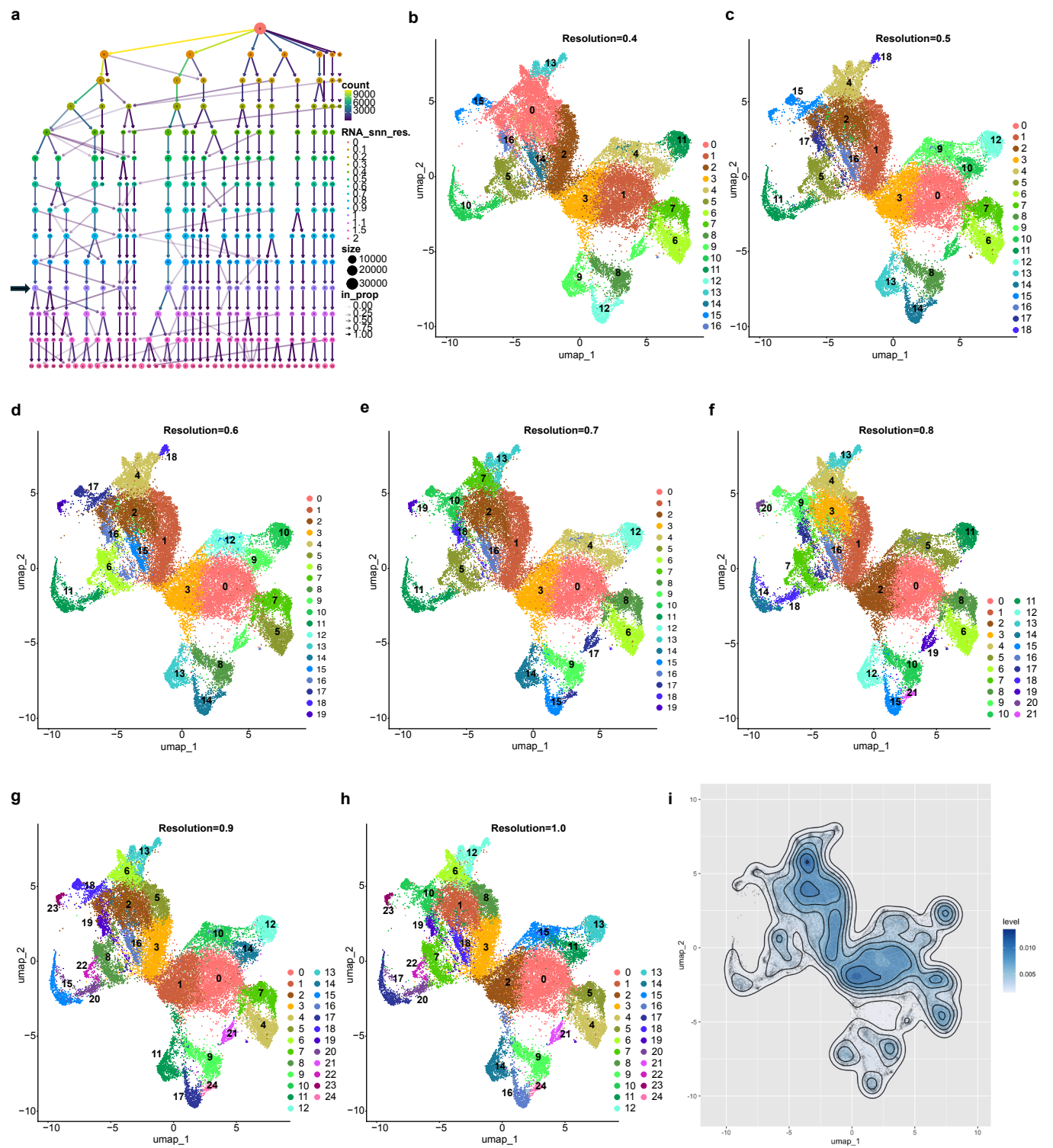

Supplement: Supplementary file 4 — Clustree output and Density plot of the integrated nTS dataset. (a) Output from clustree for the different clustering resolutions using integrated nTS dataset. In this dataset, the resolution was set to 1.0 (indicated by arrowhead). (b-h) UMAP plots of the integrated nTS dataset from Resolution 0.4 to Resolution 1.0 (see Supplementary Note 1 for details). (i) Density UMAP plot of the integrated dataset. [file 41586_2024_7608_MOESM4_ESM.pdf]
